# Variations in Total Phenolic, Total Flavonoid Contents, and Free Radicals’ Scavenging Potential of Onion Varieties Planted under Diverse Environmental Conditions

**DOI:** 10.3390/plants11070950

**Published:** 2022-03-31

**Authors:** Nusrat Bibi, Munir H. Shah, Nadeem Khan, Abdulrahman Al-Hashimi, Mohamed Soliman Elshikh, Akhtar Iqbal, Shakeel Ahmad, Arshad Mehmood Abbasi

**Affiliations:** 1 Department of Environmental Sciences, COMSATS University Islamabad, Abbottabad 22060, Pakistan; nusratniazi@ymail.com (N.B.); akhtariqbal@cuiatd.edu.pk (A.I.); 2 Department of Chemistry, Quaid-I-Azam University, Islamabad 45320, Pakistan; mhshahg@qau.edu.pk; 3 Department of Breeding and Genomics, Magnus Kahl Seeds (Pty), 6A Dairy Drive Coburg North, Coburg, VIC 3058, Australia; khan_m_nadeem@yahoo.com; 4 Department of Botany and Microbiology, College of Science, King Saud University, Riyadh 11451, Saudi Arabia; aalhashimi@ksu.edu.sa (A.A.-H.); melshikh@ksu.edu.sa (M.S.E.); 5 School of Environment, Tsinghua University, Beijing 100048, China; ahmad18@mails.tsinghua.edu.cn

**Keywords:** phenolic, flavonoid, antioxidant activity, onion, climate, soil, environment

## Abstract

Genetic diversity and Agro-climatic conditions contribute significantly to the agronomic and morphological features of the food plant species, and their nutraceutical potential. The present study was intended to evaluate the impact of growing conditions on total phenolic and total flavonoid contents, and in vitro antioxidant potential in the bulbs and leaves of onion varieties planted under diverse environmental conditions. Standard analytical methods were used to quantify total phenolic content (TPC), total flavonoid content (TFC), and free radicals’ scavenging/antioxidant capacity. The impact of climatic and soil conditions was assessed using statistical tools. In general, onion varieties cultivated at three different locations viz. Kalar Kahar, Lahore and Swabi exhibited significant variations in TPC and TFC, and antioxidant activities. The bulbs and leaves of Mustang (V1) variety planted at Lahore and Swabi had significantly (*p* < 0.05), high levels of TPC (659.5 ± 6.59, and 631.1 ± 8.58 mg GAE/100 g, respectively). However, leaves of Red Orb (V2) and bulbs of Mustang (V1), and Golden Orb (V6), harvested from Kalar Kahar depicted the highest concentration of TFC (432.5 ± 10.3, 303.0 ± 6.67, and 303.0 ± 2.52 mg QE/100 g DW, respectively). Likewise, bulbs of V1 planted at Kalar Kahar, Lahore and Swabi exhibited maximum inhibition of DPPH, ABTS, and H_2_O_2_ radicals (79.01 ± 1.49, 65.38 ± 0.99, and 59.76 ± 0.90%, respectively). Golden Orb (V6) harvested from Lahore had the highest scavenging of OH radical (67.40 ± 0.09%). Likewise, bulbs of V1 variety planted at KalarKahar and Swabi had significant capacity to scavenge ferric ions (415.1 ± 10.6 mg GAE/100 g DW), and molybdate ions (213.7 ± 0.00 mg AAE/100 g DW). Conversely, leaves of Amazon (V8), planted at Lahore and Swabi depicted significant levels of DPPH, ABTS, H_2_O_2_ radical scavenging (90.69 ± 0.26, 63.55 ± 1.06, 51.86 ± 0.43%, respectively), and reduction of ferric ions (184.2 ± 6.75 mg GAE/100 g DW). V6 leaves harvested from Lahore and that of Super Sarhad (V3) from Swabi showed the highest inhibition of OH radical (61.21 ± 0.79%), and molybdate ions (623.6 ± 0.12 mg AAE/100 g DW), respectively. Pearson correlation and principal component analysis revealed strong relationships of climatic conditions, soil properties and elevation with TPC, TFC and free radicals’ scavenging potential in the bulbs and leaves of onion varieties. The variations in the total phenolic and flavonoid contents, and antioxidant potential of different varieties, and their associations with climatic and soil factors revealed the complexity of the growing conditions and genetic makeup that imposed significant impacts on the synthesis of secondary metabolites and nutraceutical potential of food and medicinal plant species.

## 1. Introduction

Phenolic compounds are among the most substantial secondary metabolites, which are responsible for the astringency and pigmentation in plants, and act as protective agents against insects, parasites and ultraviolet (UV) radiations [1,2]. In addition, these compounds contribute efficiently in improving stress tolerance capacity in plants; play crucial role in maintaining redox-homeostasis, as well as protect the biological systems against oxidative stress [3]. It has also been reported that induction of specific metabolites influenced by the acclimation of plants to light environment may emphasize the environment-induced biochemical responses associated with the significant plasticity of phenyl propanoids metabolism [4]. In humans, phenolic acids such as gallic acid, ferulic acid, p-cumeric acid, caffeic acid etc. are intensely associated with different bioactivities mainly antimicrobial, antioxidant, anti-proliferative, anti-inflammatory, and anti-cancer for sustaining good health. In addition, phenolic acids are also being extensively used in pharmaceutical and nutraceutical industries [5]. Likewise, flavonoids viz. flavonols, flavanols, isoflavonoids, flavanones, anthocyanins and flavones represent the most ample classes of polyphenols, which are widely distributed in vegetables, fruits, medicinal herbs, and grains etc. [6,7]. In plants, flavonoids play key role in free radicals’ scavenging, mediate auxin transport, serve as signaling molecules, and involve in defense mechanisms against parasites’ attacks, and various environmental stresses [8,9,10]. Moreover, these compounds also exhibit various pharmacological and biological activities, such as anti-cancer, anti-inflammatory, anti-allergic, anti-mutagenic, antioxidant, and enzyme-regulating activities [11].

Consumption of natural antioxidants contributes significantly to the prevention of various diseases caused by oxidative stress in humans. Different types of free radicals like reactive oxygen species (ROS) and reactive nitrogen species (RNS), are generated as a result of normal cellular metabolic activities, due to UV-radiations, and various pollutants [12]. These ROS and RNS cause oxidative stress by damaging proteins, DNA molecules, carbohydrates and lipids, thus leading to aging and many degenerative disorders [13]. Living cells have well established antioxidant system to control the free radicals and lipid peroxidation, and to regulate the oxidative-antioxidative balance [14]. But, in order to scavenge ROS and RNS, the consumption of plant-based natural antioxidants, particularly polyphenols, carotenoids and vitamins is essential [15]. Consequently, a flourishing tendency originated to enrich and improve the human diet with foods containing high content of natural antioxidants. Human intervention trials, and epidemiological studies have emphasized the importance of diet rich in health beneficial secondary metabolites, specifically against the onset of metabolic disorders, chronic and non-communicable diseases including cardiovascular disorders, inflammation, ageing and various types of cancers [16,17].

Vegetables and fruits are excellent sources of natural antioxidants like polyphenols, vitamins, carotenoids etc. [18]. The genus *Allium* comprises more than 700 species, including onion, chives, garlic, shallot, and leeks. These species are different in form, taste and colour [19], and have largely been consumed as spices, vegetables, and medicines since pre-historic times [5]. *Allium cepa* L. (onion/Piyaz), is one of the most important, and health beneficial vegetable crop cultivated on a large scale throughout the world (in >175 countries), mainly in Asia [20], and is ranked second after tomato [21]. Onion is a biennial herb belonging to the family Amaryllidaceae/Alliaceae, with bulbous, scented bulbs having various shapes and skin colour. In 2020, estimated global production of onion was around 400,000 tonnes [20], which was 25% more than the past decade [22]. China is the leading country in term of area of onion cultivation and its annual production, followed by India, USA, Iran, Egypt, Turkey, and Russia, while Pakistan ranked 8^th^ in the world with 1939.6 thousand tonnes of production [23].

Onion is a versatile vegetable crop, which is consumed in a number of ways like processed products, as fresh salad, and is cooked with other animal and plant based foods [24]. Onion bulbs and leaves are rich source of nutrients such as vitamins (folic acid, C, and B6), proteins, carbohydrates, sugars (fructose, glucose, arabinose, galactose), and minerals viz. Fe, Ca, and S [25]. Bulbs and leaves of onion are rich sources of polyphenolic compounds [26,27]. More than 25 different types of flavonoid compounds (quercetin monoglucoside, quercetin diglucosides, quercetin aglycone, isorhamnetin, isorhamnetin monoglucoside; kaempferol, catechin and rutin), and anthocyanins (i.e., peonidin, pelargonidin and cyanidin) have been reported in the bulbs and leaves of onion [26,28,29,30,31,32,33]. Among phenolic acids, gallic acid, ferulic acid, syringic acid, protocatecheuic acid, chlorogenic acid, vanillic acid, coumeric acid, cinnamic acid, and benzoic acid have been quantified in the bulbs and leaves of onion varieties [26,27,34]. Epidemiological studies have suggested that regular ingestion of onion helps to decrease the risk of cardiovascular diseases, and certain types of cancers [35,36]. Moreover, anti-diabetic, anti-infalammotry and anti-atherogenic properties have also been reported in the bulb of onion [37]. Therefore, health benefits of onion are ascribed to the fact that it contains biologically active phyto-molecules, including anthocyanins, thiosulfinates, and phenolics [38].

Various environmental factors (air, water, soil, temperature, precipitation, elevation), and genetic variation among the plant species or within the individuals of same species influence extensively on the synthesis and concentration of phytochemicals and their bioactive potential [39,40]. In *Eucommia ulmoides* [41], a strong positive correlation between altitude and total flavonoid content, indicated the impact of elevation on the synthesis of secondary metabolites. Likewise, development of the phytochemicals by temperature stress is an indication of self-protection mechanism in the plant species [35], although, in some plants temperature is positively associated with the concentration of bioactive compounds. However, mostly increase in the phenolic content was observed at low temperature. For instance, a substantial increase in the concentration of total phenolic was reported in *Solanum tuberosum* and *Juglans regia* grown in the low temperature zones [42,43].

Pakistan is a place of high mountainous areas including agricultural plains and costal lines with diverse agro-climatic conditions. Therefore, various types of crops, vegetables, fruits and grains are cultivated throughout the country. Like other food crops, different varieties of onions (i.e., Desi red in Punjab, Pulkara in Sindh, Swat-1 in Khyber Pakhtunkhwa, and SariabSurkh and Chiltan-89 in Baluchistan), are cultivated in different parts of the country as commercial and kitchen garden crop. However, to the best of our knowledge, there is no comprehensive study on total phenolic and flavonoid contents, and in vitro free radicals’ scavenging or antioxidant potential in onion varieties cultivated under diverse agro-climatic conditions. In this context, the present study was mainly focused on the comparative assessment of total phenolic, total flavonoid contents, and antioxidant potential in the bulbs and leaves of onion varieties. In addition, the effects of growing conditions on TPC, TFC, and antioxidant properties of onion varieties planted under diverse growing conditions were also evaluated. In our view, the impact assessments of growing conditions, and genetic diversity on chemical composition and bioactive potential of food crops are important measures to acquire the best variety or species, specifically enriching health beneficial natural antioxidants.

## 2. Materials and Methods

### 2.1. Sampling Sites, Samples’ Collection and Processing

**Figure 1:**
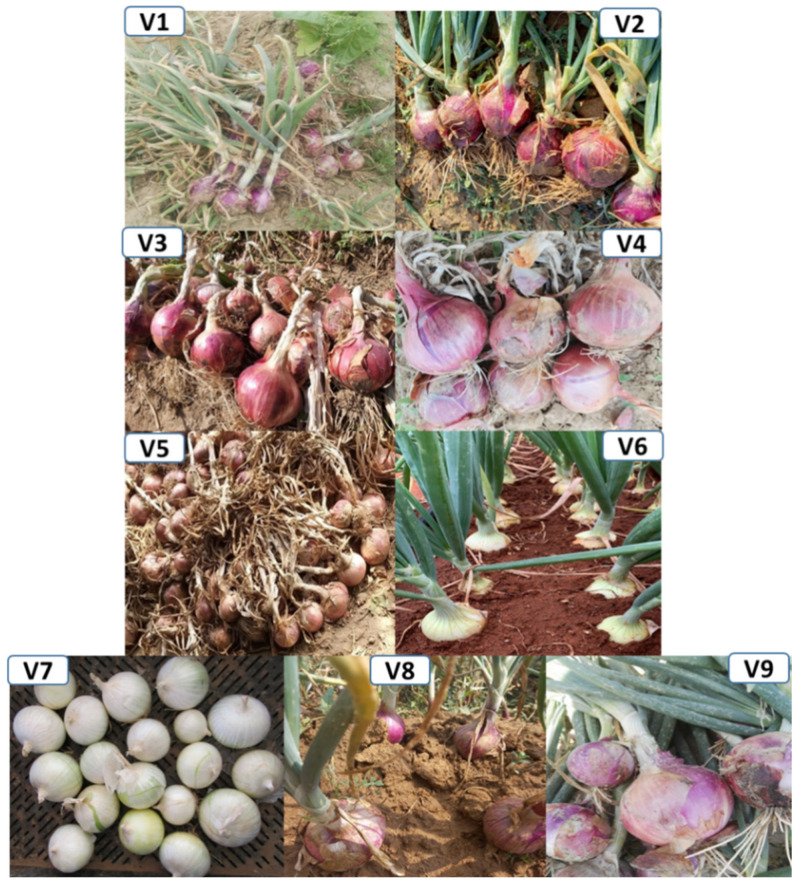
Snapshots of onion varieties. Mustang (**V1**), Red Orb (**V2**), Super Sarhad (**V3**), Red Flame (**V4**), Pulkara (**V5**), Golden Orb (**V6**), White Pearl (**V7**), Amazon (**V8**), Zeus (**V9**).

**Figure 2:**
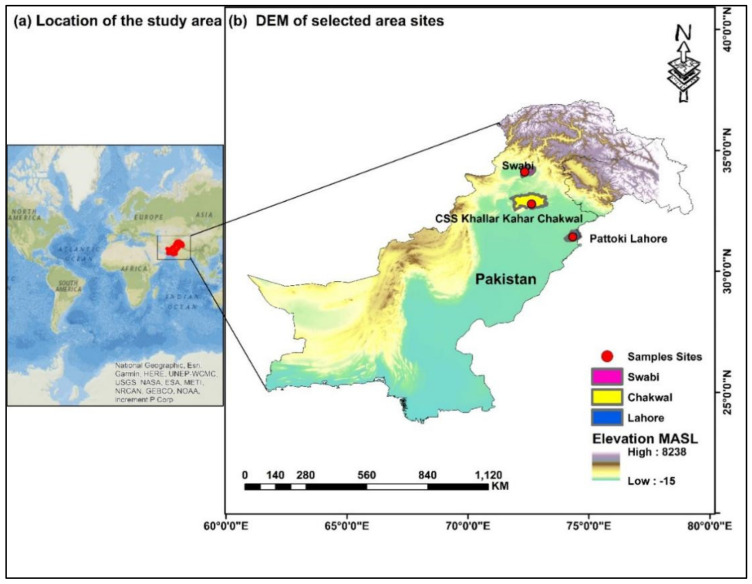
Location map showing plantation sites of onion varieties.

**Figure 3:**
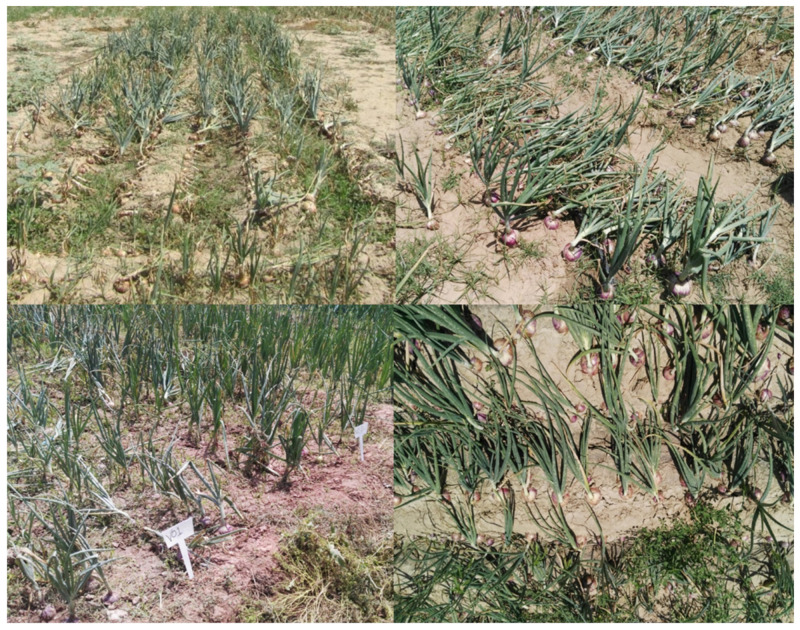
Plantation of onion varieties in the study areas.

A total of nine varieties of onion viz. Mustang, Red Orb, Super Sarhad, Red Flame, Pulkara, Golden Orb, White Pearl, Amazon and Zeus (V1–V9) were studied (Figure 1). All the varieties are enlisted in Federal Seed Certification and Registration Department (FSC&RD). The seeds of these varieties were collected from Magnus Kahl Seed-Pakistan. Nursery was raised in plastic trays under controlled conditions. Seedling of all studied varieties were transplanted in the first week of January 2017 at three different sites, namely KalarKahar (KK), Lahore (L) and Swabi (S), as illustrated in Figure 2. Three replications of each variety were planted by following “Randomized Complete Block Design (RCBD)” for nursery transplantation. Seedlings were transplanted to field in ridges with plant to plant distance of 12 cm and row to row distance of 30 cm (Figure 3).

The Barani land in Kalar Kahar is a semi-hilly area with an average elevation of 401.6 m, is located at 72°42′ E and 32°46′ N. The northern irrigation plains of Lahore are located at 74°21′ E and 31°31′ N with an average elevation of 213.0 m in the Punjab province of Pakistan [44]. Swabi is a part of northern dry mountain areas of Khyber Pakhtunkhwa (KP) province of Pakistan and is located at 34°7′ N and 72°28′ E with average elevation of 706.0 m between the Kabul and Indus Rivers [45].

Fresh leaves and bulbs were collected randomly (both at their full maturity stages) from each location in replication. Leaves were collected in May, while bulbs were harvested in late June and July. After the harvesting, samples were labelled and transferred to analytical chemistry laboratory at Quaid-I-Azam University Islamabad, Pakistan and COMSATS University Islamabad, Abbottabad Campus, Pakistan. All samples were properly washed with tap water, followed by distilled water. Bulb samples were sliced, and leaves were cut into small pieces separately, and then air dried in shade at room temperature [45]. Dried samples were crushed and grinded to fine powder distinctly, using electric grinder and stored in pre-cleaned and labelled bottles in refrigerator until further analysis.

### 2.2. Extraction

Extraction of bulbs and leaves samples was carried out by conventional solvent extraction method as described earlier by Abbasi et al. [46]. In short, precisely weighed sample (~0.5 g of each bulb and leaves of all varieties harvested from different sites), were added in conical flasks. Then, 20 mL of chilled acetone (80%) was added in each flask, and all samples were shaken overnight at room temperature using orbital shaker. Homogenates were centrifuged at ~4000× *g* m/s for 15 min, and supernatants were collected in accurately labeled flasks. The extraction process was repeated thrice, and supernatants were pooled in respective flasks, and all extracts were stored at 4 °C for further analysis.

### 2.3. Quantification of Total Phenolic and Total Flavonoid Contents

To quantify total phenolic content (TPC) in the acetone extracts of onion bulbs and leaves, Folin-Ciocalteu method was used as explained earlier [47], with some modifications. Briefly, 0.5 mL extract of each sample was added in test tubes, followed by the addition of 15 times diluted Folin-Ciocalteu reagent (2.5 mL in each sample) and 7.5% Na_2_CO_3_ (2 mL). The mixture was shaken vigorously and incubated in the dark for 90 min at room temperature. Absorbance of each sample was measured at 760 nm against blank using UV-spectrophotometer. Gallic acid was used as a standard and concentration of TPC in each sample was expressed as milligram Gallic acid equivalent in hundred grams of samples (mg GAE/100 g) on dry weight basis. Data for triplicate analysis were presented as mean ± SD.

Total flavonoid content (TFC), was also estimated using modified method of Lin et al. [47]. Briefly, 5 mL of sample extract, and 0.3 mL sodium nitrite (5%) were mixed thoroughly in properly labeled test tubes for 5 min. Subsequently, 0.3 mL of aluminum chloride (10%) was mixed, followed by the addition of sodium hydroxide (2 mL), after 6 min to stop the reaction. Absorbance was immediately measured at 510 nm against blank using UV-spectrophotometer. Quercetin was used as standard and average concentration of TFC for triplicate analysis was represented as milligram Quercetin equivalent per hundred grams of samples (mg QE/100 g) on dry weight basis. Data were presented as mean ± SD.

### 2.4. Free Radicals’ Scavenging Assays

Different assays such as DPPH, Ferric ion reducing antioxidant power (FRAP), Phosomolybdenium complex assay (PMA), ABTS, hydroxyl (OH), and hydrogen peroxide (H_2_O_2_) radicals’ scavenging assays were used to evaluate the free radicals’ scavenging or antioxidant potential in the bulbs and leaves of onion varieties cultivated under diverse growing conditions.

#### 2.4.1. DPPH Assay

Previously modified method of Chen et al. [48], was used to determine DPPH radical scavenging potential in the bulbs and leaves of onion varieties. Precisely, 1 mL of each sample’s extract, and 2.5 mL of DPPH solution (0.1 mM), were mixed in labeled test tubes, and incubated in dark for 30 min at room temperature. The absorbance was measured at 517 nm, and percentage inhibition of DPPH radical was calculated by the formula:

(1) $$\mathrm{Inhibition}\;\left(\%\right)=\frac{\left(\mathrm{Ablank}-\mathrm{Asample}\right)}{\left(\mathrm{Ablank}\right)}\times 100$$

#### 2.4.2. Ferric Ion Reducing Antioxidant Power (FRAP) Assay

Ferric ion reducing capacity in onion bulb and leaf extracts was estimated according to the method described previously by Hazra et al. [49] with some minor changes. In short, 1.0 mL each of the extract was blended with 1.0 mL (0.2 M) phosphate buffer (pH 6.6), and 1 mL potassium ferricyanide (0.1%). The reaction mixture was incubated in a water bath at 50 °C for 20 min; then, 2 mL trichloroacetic acid (10%) was added. Subsequently, an aliquot of 1 mL from upper side of solution was diluted with distilled H_2_O, and 0.01% ferric chloride solution (1 mL). This mixture was kept at room temperature for 20 min before reading absorbance at 700 nm against blank. Gallic acid was used as a positive control. Ferric ion reducing potential was expressed as mg GAE/100 g on fry weight basis for triplicate analysis.

#### 2.4.3. Phosphomolybdenum Complex Assay (PMA)

The phosphomolybdenum complex assay as reported by Prieto et al. [50], was used to calculate the total antioxidant capacity in test samples. In short, aliquot of 1 mL of sample extract was mixed with 3.3 mL freshly prepared reagent solution containing 28 mM/L sodium phosphate, 0.6 M/L sulphuric acid, and 4 mM/L ammonium molybdate. The mixture was incubated in water bath for 90 min at 95 °C, and absorbance was measured after cooling at 695 nm against blank. Inhibition of molybdate ions was expressed as milligram ascorbic acid equivalent per 100 g dry weight (mg AAE/100 g, DW) for triplicate analysis, and data were presented as mean ± SD.

#### 2.4.4. ABTS Radical Scavenging Assay

The ABTS radical scavenging activity was assessed using the method as explained previously [51]. Equal volume of ABTS solution (7 mM) was added into K_2_S_2_O_8_ (2.5 mM). Reaction mixture was incubated in the dark for 12–16 h, and a dark green solution was obtained. This solution was diluted with ethanol (50%), to obtain absorbance at 734 nm of 0.700 ± 0.02. Subsequently, 2 mL of sample extract was mixed consistently in 8 mL diluted ABTS solution. The reaction mixture was kept for 10 min and absorbance was recorded against a blank solution at wavelength of 734 nm using UV-spectrophotometer. The percentage inhibition of ABTS radical in test samples was estimated by Equation (1).

#### 2.4.5. Hydroxyl Radical (OH) Scavenging Activity

The OH radical scavenging capacity in the bulbs and leaves of onion varieties was determined by the method of Wenli et al. [52], which was based on Fenton reaction. Briefly, 2 mL of samples’ extracts were transferred to the labelled test tubes. Then in each sample, 0.04 mL ferrous sulphate (0.02 M), 2.0 mL of phosphate buffer (0.2 M/pH 7.2), and 1 mL (0.04 M) 1, 10-phenanthroline were added. Afterwards, the Fenton reaction was started by mixing 0.1 mL H_2_O_2_ (7 mM). The reaction mixture was incubated at room temperature for 5 min before taking absorbance at 560 nm. Percentage scavenging of OH radical was calculated by Equation (1).

#### 2.4.6. Hydrogen Peroxide (H_2_O_2_) Scavenging Activity

The H_2_O_2_ radical inhibition potential was estimated following the previously reported method [53]. In short, 2.0 mL sample extract was diluted with 1.2 mL of hydrogen peroxide solution (40 mM), prepared in the phosphate buffer (0.1 M/pH 7.4). The reaction mixture was incubated at room temperature for 10 min, and absorbance was measured at 230 nm by UV-spectrophotometer against a blank. The percentage scavenging of H_2_O_2_ was computed by Equation (1).

### 2.5. Climate Data

The coordinates viz. altitude, latitude, longitude, and elevation of each sampling site was taken using Global positing system (GPS), following the method of Liu et al. [54]. The data on temperature, rainfall, relative humidity, surface pressure, dew/frost point and wind speed from sowing to harvesting period of each location were provided by the Pakistan meteorological department (PMD), Islamabad-Pakistan.

### 2.6. Analysis of Soil Properties

To study the effects of soil properties of plantation sites on the concentration of TPC, TFC, and antioxidant activities in the bulbs and leaves of onion varieties, soil samples were collected randomly from each location. Composite sampling strategy as reported previously [55], was adopted. Approximately, 10–15 sub-soil samples were collected from each site, at 0–15 cm depth with the help of a spade. All sub-samples were blended subsequently into composite samples (~1 kg) to achieve homogeneity. The composite samples were spread on plastic trays and unwanted materials such as grass, stones, and gravels were removed. Cleaned samples were air dried for 48 h, and then kept in an electric oven at 70–80 °C for 48 h to attain constant weight. Finally, dried samples were ground, and passed through a sieve (6 mm). Then they were added in clean and labelled polythene bags, and kept in the desiccators till further analysis.

The soil pH and electrical conductivity (EC), were determined following the procedure described by Wang et al. [56]. Briefly soil and water in the ratio of 1:2.5 (*w*/*v*), were mixed carefully in conical flasks, and solutions were left for 16 h for stabilization. Then pH and EC were measured using pH and EC meters.

Quantification of organic matter (OM) content in the soil samples was done using loss on ignition method as reported by Nelson and Sommers [57]. Briefly, 25 g of soil samples (wet soil) of each location, was added in labeled china dish, and weight was recorded. These samples were combusted at 550 °C for 2 h in muffle furnace. Subsequently, change in the weight before and after ashing was used to determine the OM content. First, the percentage of soil volatile solids (VS) was calculated by the following formula:

(2) $$\mathrm{VS}\left(\%\right)=\frac{\mathrm{Wet}\;\mathrm{Soil}-\mathrm{Dry}\;\mathrm{Soil}}{\mathrm{Wet}\;\mathrm{Soil}}\times 100$$

where: VS represents volatile solids and moisture, Wet soil is the weight of soil before combustion and Dry soil is the soil weight after combustion.

Due to combustion at high temperature volatile solids like organic matter (in the form of CO_2_), and moisture content get evaporated. Then by dividing the total percentage of volatile solids with factor 1.8, we get the total organic matter (OM), using following formula:

(3) $$\mathrm{OM}\;\left(\%\right)=\left(\mathrm{VS}\%\right)/1.8$$

### 2.7. Statistical Analysis

All the data were analysed statistically using various softwares. Descriptive analysis (mean, standard deviations, and standard errors) was done in MS Excel tools. Variations between different variables were assessed by ANOVA test. Impacts of growing conditions on TPC, TFC, and antioxidant potential were evaluated by “Pearson’s correlation test using SPSS-13.0 (PSS Inc., Chicago, IL, USA)”, and Past 326b. Principal component analysis (PCA), following varimax normalized rotation method was also carried out by SPSS-13.0. All the data were expressed as mean ± SD and presented in graphical format with the help of Sigma Plot-V12.5, and Graph Pad Prism 8.0.1.244.

## 3. Results and Discussion

### 3.1. Variations in the Total Phenolic and Total Flavonoid Contents

It is evident that, synthesis of secondary metabolites, specifically phenolic and flavonoid contents is mainly affected by various environmental factors such as day length, intensity of light, temperature, concentration of nutrients, and water in the soil [58,59]. Measured levels of TPC and TFC in the bulbs and leaves of onion varieties planted at three localities having different growing conditions are presented in Table 1. On the whole, bulbs of Mustang variety (V1), planted at Kalar Kahar and Lahore, and Red Orb (V2), at Swabi had significantly (*p* < 0.05), higher levels of TPC, compared to the other varieties. At Kalar Kahar maximum amount of TPC was in the bulbs of Mustang variety (V1), followed by Golden Orb (V6), Zeus (V9), Super Sarhad (V3), and Red Orb (V2). However, White Pearl (V7) had the lowest concentration. In addition, there was no significant difference (*p* > 0.05), in the TPC of V2, V3, and V9 varieties. At Lahore, TPC was highest in the bulbs of V1, followed by V2, V6 and Amazon (V8). But in Red Flame (V4), TPC was not significantly different from V3 and V8 varieties. At Swabi, V2 bulb contained elevated level of TPC, followed by V1 and V6. However, there was no significant difference in the phenolic content of V3, V4, V7, V8 and V9 varieties. Comparatively, average concentrations of TPC in the bulbs of all varieties planted at three locations were analogous to previously reported levels in different varieties of onion cultivated in Korea [60], Spain [61] and China [62]. Our findings revealed that, leaves of V1, V8 and V4 varieties planted at Swabi, Lahore and Kalar Kahar, respectively had significantly high (*p* > 0.05), levels of TPC, compared to other varieties. Conversely, the lowest concentration of TPC was calculated in the leaves of Pulkara (V5) and White Pearl (V7) varieties. Relatively, average concentrations of TPC in the leaves of onion varieties at Kalar Kahar, Lahore and Swabi were corresponding to a previous report [63].

In the bulbs of onion varieties, TFC ranged from 53.29 ± 1.42 to 303.0 ± 6.67 mg QE/100 g at Kalar Kahar, from 31.86 ± 1.43 to 230.3 ± 3.78 mg QE/100 g at Lahore and from 28.95 ± 1.40 to 157.7 ± 0.00 mg QE/100 g at Swabi (Table 1) on dry weigh basis. Relatively, V1 and V6 varieties planted at Kalar Kahar, and V1 and V2 varieties planted at Lahore and Swabi showed maximum levels of TFC, while the lowest levels of TFC were in the bulbs of V7 variety planted at these locations. Leaves of V4 and V2 varieties harvested from Kalar Kahar had maximum TFC. At Lahore and Swabi, V2 and V8 varieties showed maximum TFC (Table 1). However, V5, V7 and V9 varieties contained the lowest concentration of TFC at Kalar Kahar, Lahore and Swabi, respectively. Reasonably, TFC in the bulbs of all varieties were higher than those reported earlier by Zhou et al. [62] from China. However, TFC in the leaves of onion had rarely been reported so far.

**Figure 4:**
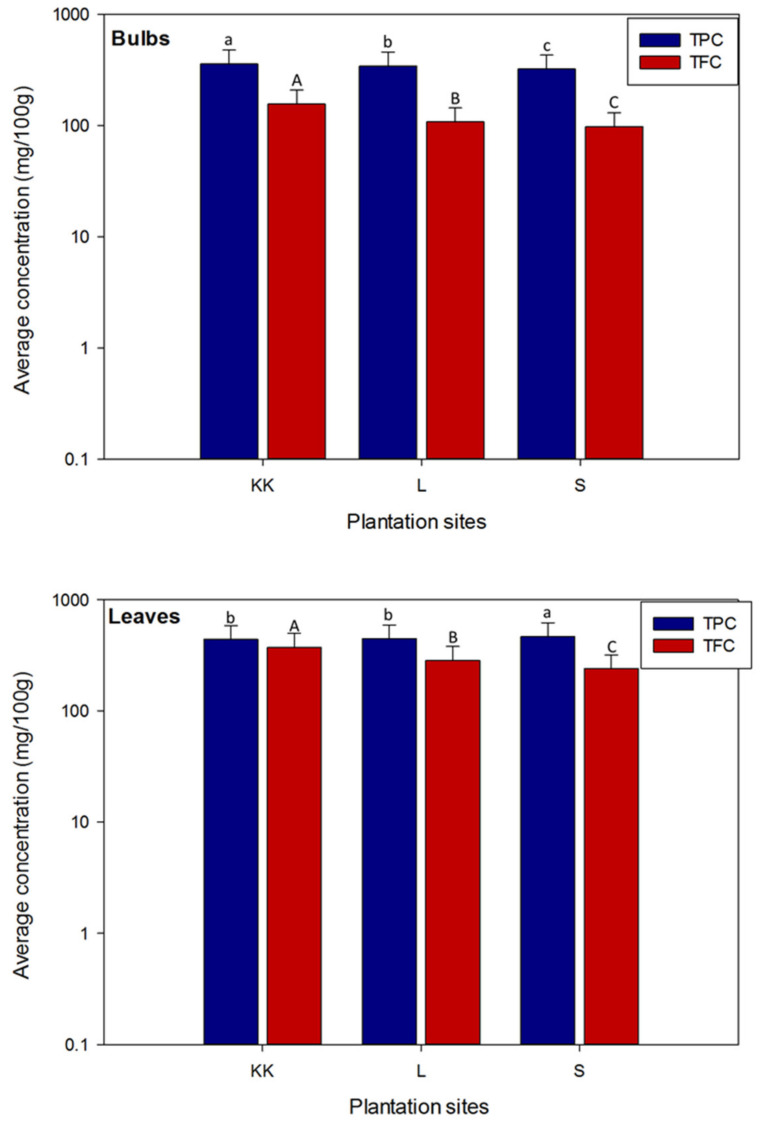
Comparative assessment of TPC and TFC in the bulbs and leaves of onion varieties at different plantation sites. Different letters (a–c/A–C), indicate significant differences (*p <* 0.05) in TPC and TFC.

Mean concentrations of total phenolic and total flavonoid contents in the bulbs and leaves of onion varieties planted at three different locations are illustrated in Figure 4. Our findings revealed that mostly onion leaves contained more TPC and TFC, compared to the bulbs. In the bulb samples, average concentration of TPC and TFC was significantly higher (*p* < 0.05), in onion varieties planted at Kalar Kahar (357.0 ± 119 mg GAE/100 g, 155.9 ± 52.0 mg QE/100 g, respectively), compared to the Lahore and Swabi. In the leaves’ samples, average concentration of TPC was significantly (*p* < 0.05) higher in onion varieties cultivated at Swabi (465.3 ± 155.0 mg GAE/100 g, DW), compared to the other locations. However, TPC was not significantly different in the leaves of onion varieties planted at Lahore and Kalar Kahar. Conversely, mean concentration of TFC was considerably high in the leaves of onion varieties planted at Kalar Kahar, followed by Lahore and Swabi (371.5 ± 123.8, 284.3 ± 94.8 and 239.5 ± 79.8 mg QE/100 g, DW, respectively). Similar trend was noted in the bulbs of onion varieties, where mean concentration of TFC was significantly (*p* < 0.05) higher in onion varieties planted at Kalar Kahar (155.9 ± 52.0 mg QE/100 g, DW), followed by the Lahore and Swabi locations (107.9 ± 36.0 and 97.40 ± 32.5 mg QE/100 g, DW, respectively).

### 3.2. Disparity in Free Radicals’ Scavenging/Antioxidant Potential

It is not appropriate to come to conclusion about the antioxidant potential in plant extracts based on single test model [64]. Because, up to now, there is no well optimized, and standardized assay that can provide an inclusive image of the antioxidant capacity in test samples [65]. It has been reported that for testing antioxidant activity/capacity of natural compounds in the targeted samples, multi in vitro antioxidant test models that measure the electron or hydrogen atom transfer from antioxidant compounds to free radicals (reactive oxygen or reactive nitrogen species), must be used [66,67,68]. Moreover, quantification of total phenolic, total flavonoid by colorimetric methods, and determination of antioxidant activity using in vitro antioxidant assays are very useful approaches to evaluate the health beneficial potential, and for the quality control of food materials, and natural products [69,70,71]. Therefore, in the present study, free radicals’ inhibition or antioxidant potential in the bulbs and leaves of nine varieties of onion, planted at three diverse locations was evaluated using various assays viz. DPPH, OH, H_2_O_2_, ABTS, FRAP and PMA.

#### 3.2.1. DPPH Radical Inhibition Potential in the Bulbs and Leaves of Onion Varieties

The DPPH in vitro assay is a simple, economical, reproducible, and most frequently used method to evaluate the overall antioxidant capacity of free radical scavengers present in natural foods, fruits, vegetables etc. [66]. This assay is based on the reduction of DPPH, a stable radical to DPPHH by hydrogen atom donor such as antioxidant compounds in food substances [72]. The percentage inhibition of DPPH radical in the bulbs and leaves of onion varieties is mentioned in Table 2. Bulbs samples of Mustang (V1), and Golden Orb (V6) varieties collected from Kalar Kahar depicted significant capacity (*p* < 0.05) to scavenge DPPH radical (79.01 ± 1.49 and 78.05 ± 0.61%, respectively). At Lahore V1 and V2, while at Swabi V1 and Super Sarhad (V3) varieties exhibited maximum potential, which was significantly (*p* < 0.05) higher than other varieties. Conversely, Zeus (V9) from Lahore, and White Pearl (V7) from Kalar Kahar and Swabi showed the lowest DPPH scavenging activity. In the leaves samples, at Kalar Kahar percentage inhibition of DPPH radical was maximum in V1, V2, V3 and V5 varieties ranging from 61.77 ± 0.87 to 63.85 ± 1.23%. And there were no significant differences (*p* > 0.05), in the DPPH radical inhibition potential in the leaves of these varieties. Likewise, at Lahore V8 and V9 varieties showed maximum potential, while at Swabi V1 and V2 varieties had the highest DPPH scavenging capacity, compared to other varieties with significant difference at *p* < 0.05.

**Figure 5:**
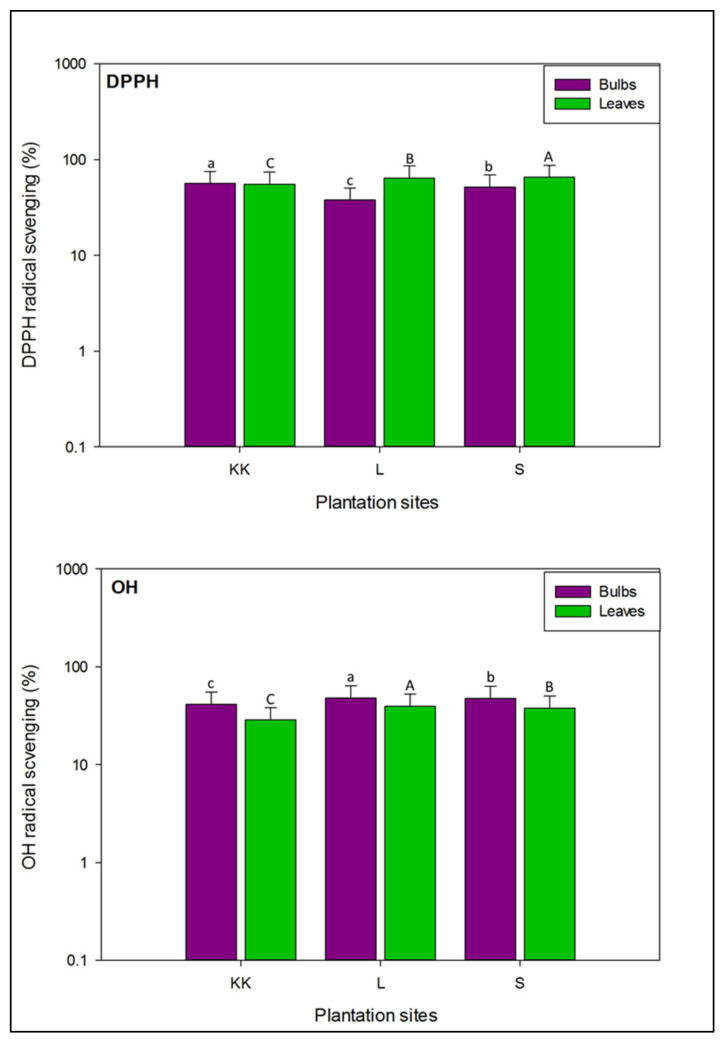
Comparative assessment of DPPH and OH radicals’ scavenging in the bulbs and leaves of onion varieties at different plantation sites. Different letters (a–c/A–C) indicate significant difference (at *p <* 0.05) in bulbs and leaves at different locations.

As illustrated in Figure 5, leaves of onion varieties had more potential to scavenge DPPH radical, compared to the bulbs. Our findings revealed that in the bulb samples, DPPH radical scavenging activity varied significantly (*p* < 0.05), in all varieties planted at three locations. The highest percentage scavenging of DPPH radical was noted in the bulbs of onion varieties cultivated at Kalar Kahar (56.55 ± 18.9%), followed by Swabi and Lahore (51.67 ± 17.2 and 37.92 ± 12.6%, respectively). In the leaves’ samples (Figure 5), DPPH activity was significantly higher (*p* < 0.05), in onion varieties cultivated at Swabi (65.55 ± 21.8%), followed by Lahore and Kalar Kahar (64.22 ± 24.1 and 55.39 ± 18.4%, respectively). On the whole, our findings revealed that bulbs of Mustang (V1), harvested from all three localities possessed maximum DPPH radical scavenging potential, while leaves of onion varieties exhibited diverse trends. In addition, average levels of DPPH radical inhibition caused by the bulbs and leaves extracts of onion varieties at three locations were analogous to previous reports [73,74,75].

#### 3.2.2. Hydroxyl Radical Scavenging Ability in Onion Varieties

Hydroxyl radical is one of the powerful reactive oxygen species (ROS) that damages living cells by reacting with phospholipid molecules present in the cell membrane [64,76]. Hydroxyl radical scavenging assay is a commonly used method to estimate the antioxidant capacity of secondary metabolites present in the fruits and vegetables against OH radical, which is produced by the disruption of hydrophilic chain [77,78]. Results showing percentage scavenging of OH radical in the bulbs and leaves of onion varieties are presented in Table 2. In the bulb samples, significantly high OH radical scavenging (*p* < 0.05) was noted in Golden Orb (V6), planted at Lahore (67.40 ± 0.09%), followed by Red Flame (V4), at Kalar Kahar and Swabi (62.13 ± 0.11 and 56.86 ± 0.29%, respectively). Conversely, Zeus (V9) had the lowest potential to scavenge OH radical. On the other hand, leaves of Golden Orb (V6) collected from Lahore showed maximum OH radical scavenging potential (61.21 ± 0.76%), followed by Mustang (V1), from Kalar Kahar and Pulkara from Swabi (51.76 ± 2.23 and 50.00 ± 0.00%, respectively), and these values were significantly varied at *p* < 0.05, from majority of varieties at three locations.

As shown in Figure 5, relatively OH radical scavenging potential was higher in the bulb samples, compared to the leaves’ samples of onion varieties planted at three different locations. In addition, there was significant difference (*p* < 0.05), in the bulbs and leaves of onion varieties collected from all locations to scavenging the OH radical. The bulbs of onion varieties cultivated at Lahore had high OH radical inhibition capacity (48.24 ± 16.1%), followed by Swabi and Kalar Kahar (47.26 ± 15.7, 41.31 ± 13.7%, respectively). Likewise, leaves’ samples (Figure 5), collected from Lahore showed maximum OH radical scavenging, followed by Swabi and Kalar Kahar (39.40 ± 13.1, 38.02 ± 12.6, 28.69 ± 9.56%, respectively).

#### 3.2.3. Hydrogen Peroxide Inhibition Capacity

Hydrogen peroxide is a major metabolite of oxygen. It is a weak oxidizing agent that produced in the body of living organisms by oxidase enzymes and activated phagocytes [58]. After crossing the cell membrane, H_2_O_2_ molecules probably react with ferrous and cuprous ions, and generate OH radicals that cause toxic effects in living organisms. Therefore, it is beneficial for cells to control the amount of H_2_O_2_ [76]. The H_2_O_2_ inhibition capacity in the bulbs and leaves of onion varieties is presented in Table 3. In the bulb samples, percentage scavenging of H_2_O_2_ radical ranged from 33.33 ± 0.82 to 53.72 ± 1.29% at Kalar Kahar, from 31.31 ± 0.74 to 55.35 ± 0.93% at Lahore and from 26.97 ± 0.87 to 59.76 ± 0.90% at Swabi. Comparatively, bulbs of V1 cultivated at Kalar Kahar, Lahore and Swabi exhibited significantly high (*p* < 0.05) scavenging of H_2_O_2_ radical at 53.72 ± 1.29, 55.35 ± 0.93, 59.76 ± 0.90%, respectively. It was noted that H_2_O_2_ inhibition potential in the bulbs of onion varieties planted at three different sites was in agreement with previously reported levels by Bankeblia [79]. In the leaves, scavenging of H_2_O_2_ radical was maximum in V8 variety, planted at Swabi (51.86 ± 0.43%) followed by V2 and V6 varieties planted at Lahore and Kalar Kahar (44.18 ± 1.63, and 40.87 ± 0.35%, respectively). The inhibition potential of these varieties was significantly different at *p* < 0.05, compared to all other varieties. However, V5 and V7 varieties had relatively lower H_2_O_2_ radical scavenging capacity.

**Figure 6:**
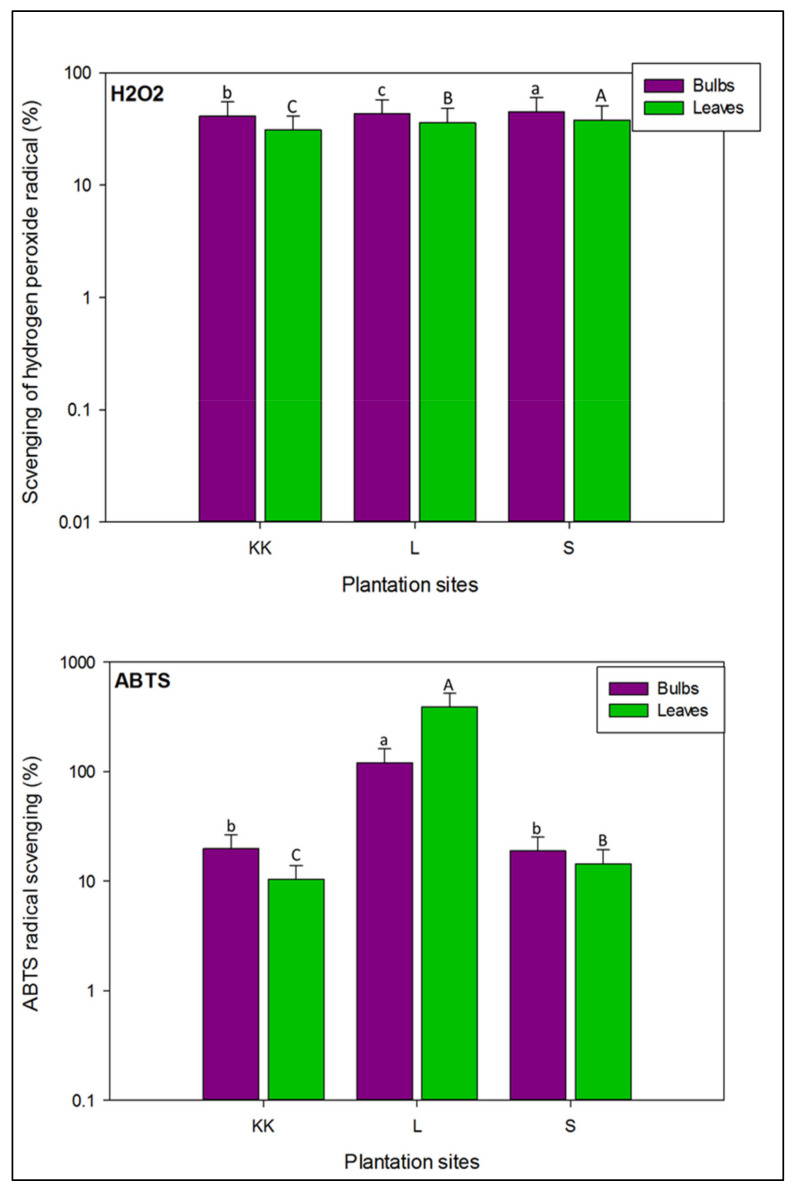
Comparative assessment of H_2_O_2_ and ABTS radicals scavenging in the bulbs and leaves of onion varieties at different plantation sites. Different letters (a–c) indicate significant difference between values at *p* < 0.05. Different letters (a–c/A–C) indicate significant difference (at *p* < 0.05) in bulbs and leaves at different locations.

As shown in Figure 6, the H_2_O_2_ radical scavenging potential was high in the bulb samples, compared to the leaves of onion varieties planted at all three cultivation sites with significant difference (*p <* 0.05). Average percentage scavenging of H_2_O_2_ radical in the bulbs of onion varieties planted at Swabi was significantly high (45.27 ± 15.1%), followed by Lahore and Kalar Kahar (43.33 ± 14.4, and 41.41 ± 13.8%, respectively). Likewise, leaves of onion varieties harvested from Swabi had maximum capacity to scavenge H_2_O_2_ radical (37.98 ± 12.7%), followed by varieties planted at Lahore and Kalar Kahar (36.25 ± 12.0 and 31.03 ± 10.3%, respectively).

#### 3.2.4. ABTS Radical Scavenging Capacity

ABTS [2, 20 azino-bis (3-ethylbenzothiazoline-6-sulfonic acid], radical scavenging assay is based on the interaction between pre-generated “ABTS^•^” radical and an antioxidant molecule [76]. ABTS assay is a useful method in evaluating the antioxidant capacity of natural and synthetic compounds soluble in the organic as well as aqueous solvents [66,80,81]. This assay is simple, time saving, cost effective, and is being extensively used over a wide range of pH to differentiate between additive and synergistic effects of various secondary metabolites in plant species [76,82]. The percentage reduction of ABTS radical in the bulbs and leaves of onion varieties is presented in Table 3. Extracts of V6, V5 and V1 bulbs harvested from Kalar Kahar showed highly significant capability to scavenge ABTS radical (32.80 ± 14.5, 31.37 ± 12.7, and 30.17 ± 2.02%, respectively). At Lahore and Swabi, V1 and V2 exhibited maximum potential (65.38 ± 0.99 and 41.92 ± 0.35%, respectively).

However, bulbs of V1, V9 and V7 planted at Swabi, Lahore and Kalar Kahar had the lowest ABTS radical scavenge potential. Relatively, ABTS radical inhibition potential in the bulbs of onion varieties was similar to previous report [66]. However, in the leaves of onion varieties, ABTS radical scavenging activity was studied for the first time. There was no significant difference in ABTS^•^ scavenging potential in the leaves of onion varieties planted at Kalar Kahar, whereas at Lahore and Swabi, V8 and V9 varieties exhibited significant potential (*p <* 0.05).

Overall, based on average levels of ABTS radical inhibition potential in onion varieties, bulbs were dominant over leaves (Figure 6). Comparatively, bulbs of onion varieties planted at Lahore showed significantly higher ABTS radical scavenging (46.32 ± 15.4%), than planted at other locations i.e., Kalar Kahar and Swabi (19.48 ± 6.61 and 18.92 ± 6.31%, respectively). However, there was no significant difference in ABTS radical scavenging observed in the bulbs of onion varieties planted at Kalar Kahar and Swabi. In the case of leaves’ samples, onion varieties planted at Lahore had maximum inhibition of ABTS radical, followed by Swabi and Kalar Kahar (47.20 ± 15.7, 14.49 ± 4.82 and 10.42 ± 3.47%, respectively), with significant difference at *p* < 0.05.

#### 3.2.5. Ferric Ion Reducing Potential in Onion Varieties

FRAP assay is a simple, frequently utilized, cost effective, and an authenticated method to study the antioxidant potential on a large scale in the body fluids, foods, and beverages. This method is useful to evaluate the impact of changing environment, post-harvest conditions, industrial processing and genetic variations on the antioxidant potential in foods and other plant materials. In addition, FRAP method also contributes in quality control assessment, product differentiation, and development [66,83,84]. In this assay, antioxidant compounds in samples (fruits, vegetables, grains etc.), reduce oxidation state of Fe^3+^ to Fe^2+^ by donating an electron [85]. Measured levels of FRAP in the bulbs and leaves of all varieties are shown in Table 4. Relatively, bulbs of V1 planted at Kalar Kahar had significantly high (*p* < 0.05) ferric ion reduction power, followed by Lahore and Swabi (415.1 ± 10.7, 373.5 ± 16.5, 271.2 ± 27.6 mg GAE/100 g, respectively) on dry weight basis. However, bulbs of Pulkara (V5), harvested from Swabi and Lahore, and of White Pearl (V7) from Kalar Kahar showed the lowest potential to reduce ferric ions. Moreover, average potential of ferric ions’ reducing power in the bulbs of onion varieties planted at three different locations was high compared to previously reported levels [73]. Leaves of V2 and V8 varieties harvested from Kalar Kahar exhibited highest ferric ion reducing power (92.41 ± 0.61 and 80.62 ± 5.21 mg GAE/100 g). Likewise, at Lahore and Swabi, the same varieties were dominating with FRAP values: 184.2 ± 6.75 mg GAE/100 g (V8), and 157.7 ± 10.1 mg GAE/100 g (V2).

**Figure 7:**
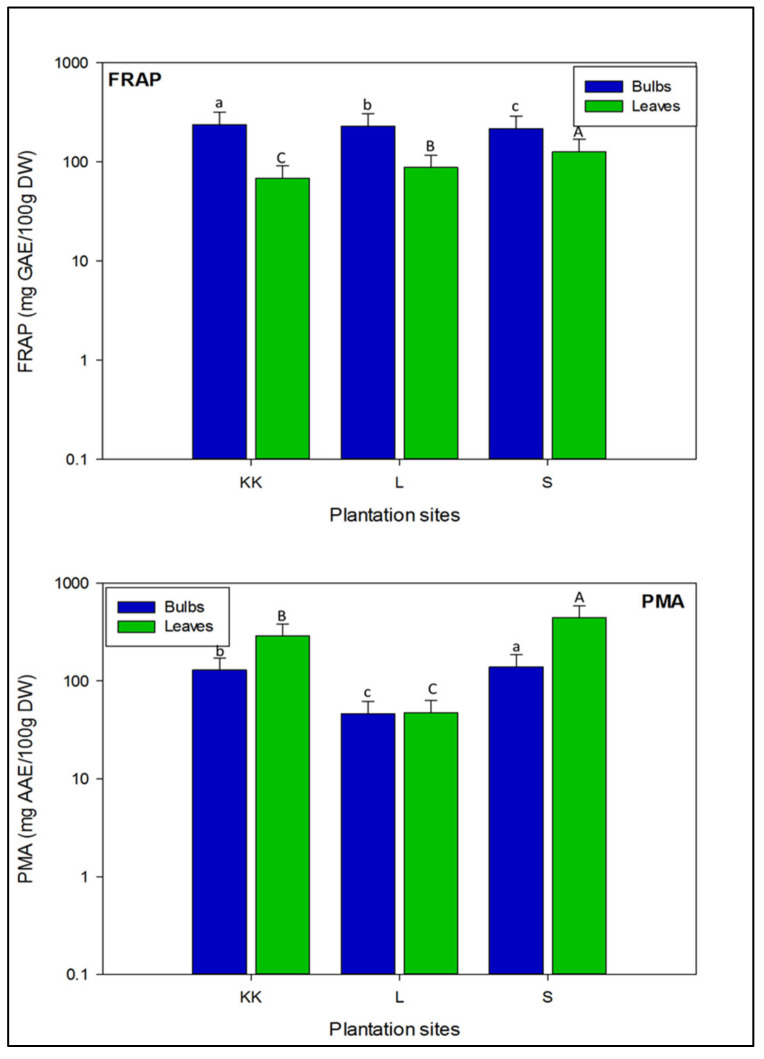
Comparative assessment of FRAP and PMA values in the bulbs and leaves of onion varieties at different plantation sites. Different letters (a–c/A–C) indicate significant difference (at *p <* 0.05) in bulbs and leaves at different locations.

As shown in Figure 7, bulbs of onion varieties had more ferric ion reduction capacity than leaves. Based on average values of FRAP, significant variations (*p* < 0.05) were noted in the bulbs and leaves of onion varieties planted at three different locations. The bulbs of onion varieties planted at Kalar Kahar showed maximum potential to reduce ferric ions (237.7 ± 79.2 mg GAE/100 g), followed by onion varieties planted at Lahore and Swabi (228.1 ± 76.0 and 216.2 ± 72.0 mg GAE/100 g, respectively). The leaves of onion varieties harvested from Swabi had significantly higher ferric ion reduction potential (126.2 ± 42.1 mg GAE/100 g), followed by those harvested from Lahore and Kalar Kahar (87.78 ± 29.2 and 68.01 ± 22.1 mg GAE/100 g, respectively). 

#### 3.2.6. Comparative Assessment of Total Antioxidant Capacity

Phosphomolybdenum complex assay (PMA), was used to estimate total antioxidant potential in the bulbs and leaves of onion varieties cultivated under diverse environment. This assay is based on the reduction of molybdate ions “Phosphate-Molybdenum (VI) to bluish green Phosphate-Molybdenum (V)”. The PMA method can be used to determine antioxidant activity in various types of samples such as cosmeceutical and pharmaceutical products, plant extracts, specifically in liphophilic solvents, vegetable oils, serum, and butter etc. [50]. Measured levels of molybdate ions reducing capacity in the bulbs and leaves of onion varieties are given in Table 4. It is evident that bulbs of V9, V6 and V1 varieties collected from Kalar Kahar had maximum potential to reduce the molybdate ions (160.7 ± 4.03, 157.0 ± 9.43, and 141.3 ± 9.14 mg AAE/100 g), with no significant difference at *p* < 0.05. At Swabi and Lahore V1 variety showed substantial potential to inhibit the molybdate ions (213.8 ± 0.00, and 194.2 ± 9.54, respectively). The leaves of V3 variety harvested from Swabi and Lahore areas showed maximum capacity to inhibit molybdate ions (623.6 ± 0.72, and 523.0 ± 0.00 mg AAE/100 g, respectively). However, at Kalar Kahar V7 had significant potential (411.6 ± 13.0 mg AAE/100 g), to reduce molybdate ions, compared to the other varieties.

As illustrated in Figure 7, average levels of molybdate ions reducing capacity were high in the leaves of onion varieties, compared to the bulbs. There were significant differences (*p* < 0.05), in the measured levels of molybdate ions inhibition in all varieties at three different sites. Comparatively, bulbs of onion varieties planted at Swabi had maximum potential to scavenge molybdate ions (139.7 ± 46.5 mg AAE/100 g, DW), followed by those planted at Kalar Kahar and Lahore (129.1 ± 43.0, and 46.32 ± 15.4 mg AAE/100 g, DW, respectively). Likewise, based on mean concentration, leaves of onion varieties harvested from Swabi exhibited significant potential (442.9 ± 147.6 mg AAE/100 g, DW) to reduce the molybdate ions, followed by the varieties planted at Kalar Kahar and Lahore (288.3 ± 96.1, and 47.20 ± 15.7 mg AAE/100 g, DW, respectively). Moreover, in the present study total antioxidant potential in the bulbs and leaves of onion varieties was higher than reported by Ola–Mudathir et al. [73] from Nigeria.

In summary, bulbs and leaves of Mustang (V1), Red Orb (V2) and Amazon (V8), harvested from three locations (Kalar Kahar, Lahore and Swabi), had maximum concentration of total phenolics and flavonoids, and showed significant free radical scavenging/antioxidant potential at all locations. It was noted that onion varieties planted at three different locations had significant disparities in the concentrations of TPC, TFC, and in vitro antioxidant activities determined by various assays. Bulbs of onion varieties cultivated at Kalar Kahar had significantly high TPC and TFC, compared to other locations, whereas leaves collected from Swabi and Kalar Kahar contained maximum TPC and TFC, respectively. In both leaves and bulbs of onion varieties OH and ABTS radicals scavenging capacity was high in varieties planted at Lahore, and reduction of H_2_O_2_ and molybdate ions was maximum at Swabi. The DPPH and FRAP activities were high in the bulbs collected from Kalar Kahar, but these activities were high in the leaves of onion varieties planted at Swabi. These findings support the assumption that change in growing environment and genetic variations between different plant species, and within the population of same species, specifically onion (*Allium cepa*) affect substantially the synthesis of phenolics, flavonoids and their bioactive potential. Moreover, our results also validate the findings of other researchers [86,87,88].

In the present study, measured levels of TPC and TFC were high in the leaves of onion varieties, compared to the bulbs. These findings specify the difference in the production, distribution and accumulation of secondary metabolites in various parts of plant species, and also within the varieties of same species as reported earlier [89]. In the present study, more concentration of TPC and TFC was noted in leaves, compared to the bulbs of onion varieties. These findings corresponded to Kabtni et al. [90], who reported that leaves were the major parts in the plant species i.e., *Medicago* that contained maximum total phenolic and flavonoid contents. There are several reports that identify relationship between exposure to UV-B radiations and higher production of polyphenols, such as in barley and Arabidopsis [91,92]. Therefore, substantial levels of phenolic and flavonoid contents in the leaves of onion varieties indicate various interactions between aerial parts of plant species and their surrounding environment. Because, changes in the atmospheric temperature, precipitation, humidity, radiation, sunlight etc. affect significantly not only the growth and development of plants, but also the synthesis of secondary metabolites and bioactive potential. And as a part of their defensive strategy, plants produce more secondary metabolites that protect them from adverse effects of environmental stresses [90]. Flavonoids, the most abundant class of phenolic compounds protect plant cells from UV radiations by accumulating in the epidermal layers of leaves. These compounds absorb light in the UV-B region, while allowing visible wavelengths to pass through the cells without any interruption [93].

Free radicals scavenging/antioxidant potential, was more in the bulbs except DPPH and molybdate ions reduction activities, which were high in the leaves of onion varieties. These findings confirm the synergistic and antagonistic role of various phytochemicals including phenolic compounds, in the bioactive potential of food and medicinal plant species [84,94]. Moreover, our results endorse the fact that underground plant parts like, roots, rhizome, bulbs etc. have more secondary metabolites, particularly polyphenols in arid and semi-arid regions as reported by Gargallo-Garriga et al. [95]. Because, in underground environment, soil composition, water availability, temperature and microbial consortia affect considerably the metabolic activities [96]. However, low temperature and scarcity of water in the underground environment may also cause significant decrease in the concentration of phytochemicals and bioactive potential in roots, bulbs, rhizome etc. [95,97].

### 3.3. Correlation Analysis

Pearson correlation coefficient matrices were applied to evaluate the relationships between mean concentrations of TPC, TFC and antioxidant activities in the bulbs and leaves of onion varieties. As demonstrated in Figure S1, TPC and TFC in the bulbs of all varieties had strong positive and negative associations with antioxidant activities. For instance, highly significant (*p <* 0.05) positive relationships (100%), were noted between TFC-ABTS, TFC-DPPH, TPC-DPPH, and TPC-FRAP, in V2, V4, V5 and V9 varieties, respectively. Likewise, >99.00% positive relations were noted between TPC-FRAP, TPC-PMA, TPC-H_2_O_2_ (V1, V2 and V3 varieties, respectively), and between TFC-PMA and TFC-ABTS in V5 and V8 varieties. However, in the bulbs of White Pearl (V7), TFC showed significantly negative association (100%, *p <* 0.05), with DPPH and FRAP assays. Similarly, >90.00% negative associations were also observed in TPC-PMA, TPC-OH, TPC-ABTS (V2, V3 and V8 varieties), and in TFC-PMA, TFC-H_2_O_2_, TFC-ABTS, TFC-FRAP (V1, V3, V5, V5 and V9 varieties). Results of correlation analysis between TPC, TFC, and antioxidant activities in the leaves of onion varieties are shown in Figure S2. The TPC exhibited strong positive associations (>90.00%), with ABTS, H_2_O_2_ and OH radicals scavenging activities in the leaves of V5, V7, V8 and V9 varieties, whereas, TFC depicted strong positive relationships (>90.00%), with ABTS, and DPPH activities in V1, V5, V6 and V7 varieties. In addition, highly significant positive (100%, *p <* 0.05) interaction was observed between TFC and OH radical scavenging potential determined in the leaves of Red Flame (V4). Conversely, TFC showed strong negative associations (>90.00%), with DPPH and OH radicals scavenging activities in V2, V3, V8 and V9 varieties. Our findings revealed that positive association of phenolic and flavonoid contents with antioxidant activities was an indication of their significant contribution in the inhibition of free radical species [98,99,100]. However, negative relations also exposed the synergistic role of other metabolites i.e., carotenoids, anthocyanins, organo-sulfur compounds, vitamins and metal antioxidants in onion, specifically [15,26,27,101,102].

### 3.4. Impact of Growing Conditions Secondary Metabolites and Antioxidant Activities

Even though, the production of secondary metabolites in plant species is mainly directed by various genetic pathways, the role of growing conditions or ecological factors (changing climate, soil properties, altitudinal variation, and day length etc.) cannot be denied [39]. Synthesis and concentration of bioactive compounds viz. hydrophilic and lipophilic antioxidants, in plant species have strong associations with changing climate. And the amount of bioactive substances in various plant parts increases or decreases significantly due to change in the growing environment [103]. Variations in temperature, humidity, precipitation, sunlight duration and intensity, solar radiations, altitude and soil properties affect considerably the expression of various genes, and ultimately affect the synthesis of secondary metabolites i.e., polyphenols and bioactive potential of plant species [47,103].

Onion is one of the main horticultural crop containing significant levels of health beneficial phytochemicals including polyphenols, organo-sulpher compounds, and polysaccharides [104]. These phytochemicals are excellent natural phyto-antioxidants, and possess substantial capacity to scavenge the free radicals’ species. With reference to TPC, TFC and antioxidant activities, significant variations were observed in the bulbs and leaves of onion varieties planted at three different locations in the present study. For instance, onion varieties cultivated at Lahore showed significant potential to scavenge ABTS and OH radicals; those planted at Swabi were more effective in the reduction of molybdate ions and H_2_O_2_ radicals, while those planted at Kalar Kahar exhibited significant reduction of DPPH and ferric ions radicals. In addition, such variations were also observed within plant part(s) viz. bulbs and leaves of the same variety. For example, bulbs of White Pearl (V7) had the lowest concentration of TFC at all three locations, but the leaves of the same variety harvested from Kalar Kahar contained maximum total flavonoid content. These findings confirm the impact of change in the growing conditions and soil properties, along with genetic diversity among different plant species or within the same species. Both genetic variations and growing environment are important factors that affect the synthesis of secondary metabolites and antioxidant potential of plants [54,105,106]. Therefore, our findings confirm that the difference in TPC and TFC, and antioxidant capacity in different varieties of onion cultivated at three different locations is mainly influenced by genetic variation among onion varieties and growing environment [107,108,109]. Moreover, concentration of phytochemicals i.e., phenolic and flavonoid compounds could be used as “biochemical markers”, to differentiate among the individuals of same populace like *Allium cepa* (onion), at species levels, as well that of different species at generic levels. Likewise, distribution of TPC or TFC in different parts of onion (leaves and bulbs), could also be used in genetic engineering for more valorization of bioactive compounds in onion and other plant species used as food or medicine.

#### 3.4.1. Impact of Climatic Conditions on Total Phenolic, Total Flavonoid Contents and Antioxidant Activities

In the present study, total phenolic and total flavonoid contents were quantified in the bulbs and leaves of onion varieties. However, due to the lack of HPLC or GC-MS we were unable to determine phenolic acids and flavonoids compounds. Based on Pearson’s correlation analysis, different climatic conditions viz. temperature, rain fall, relative humidity, dew or frost point, surface pressure, wind speed, altitude (as mentioned in Table 5), showed strong associations with total phenolic, total flavonoid, and antioxidant activities in the bulbs and leaves of onion varieties (Tables S1 and S2).

##### Impact of Temperature

As presented in Table S1, temperature exhibited diverse effects on TPC, TFC and antioxidant activities in the bulbs of onion varieties (Table S1). For example, Max.T showed highly significant (*p* < 0.01) inverse correlations with TPC and PMA in Mustang (V1), and with OH radical scavenging activity in White Pearl (V7). However, same temperature had strong positive association (*p* < 0.05) with TPC and DPPH in the bulbs of Pulkara variety (V5). Likewise, in leaves (Table S2), Max.T showed significantly negative correlations (*p* < 0.01 and *p* < 0.05), with DPPH activity in Mustang (V1) and Red Flame (V4), with FRAP activity in Golden Orb (V6), and with TPC in Zeus (V9) varieties.

Conversely, Min.T exhibited positive correlations (*p* < 0.01) with ABTS, TPC, and H_2_O_2_ in the bulbs of Golden Orb (V6), Mustang (V1) and Super Sarhad (V3) varieties (Table S1). However, strong negative relations were noted with PMA (−1.000) in Amazon (V8). Likewise, Min.T showed significantly (*p* < 0.01) positive associations with OH radical scavenging activity, and TFC in the leaves of Golden Orb (V6) and Zeus (V9), respectively (Table S2). Mean temperature (MT) showed strong negative relations (*p* < 0.05), with antioxidant activities in the bulbs and leaves of different varieties (Tables S1 and S2), such as with DPPH and OH activities in the bulbs of Amazon (V8), while with FRAP and PMA activities in the leaves of Pulkara (V5), and Mustang (V1).

These findings revealed that, overall, the temperature affected TPC, TFC, and antioxidant activities in the bulbs and leaves of onion. However, the effect of Max.T on TPC and antioxidant activities was more pronounced in both bulbs and leaves, compared to Min.T and MT. In contradiction to our findings, Kumar et al. [110] reported that antioxidant activities were more prominent in cold weather. The diverse relationships of temperature with TPC and TFC in the bulbs and leaves of onion varieties were in agreement with those reported earlier in other plant species. For instance, Boussaa et al. [111], reported strong negative relations between TPC and temperature in Pomegranate fruit. In another study, Kumar et al. [112] proposed that low temperature caused more production of phenolics and vice versa, which was in contradiction to our findings. Inconsistently, Moreira et al. [113], mentioned a positive correlation between temperature and TPC. However, Wang [114], and Liu et al. [54] reported negative correlations between phenolics, flavonoids and temperature.

Likewise, Max.T and MT exhibited highly significant negative correlations with antioxidant activities except DPPH inhibition potential in bulb samples. However, Min.T showed a positive relation with antioxidant activities both in bulbs and leaves except for OH in bulbs. Correspondingly, Wang [114] reported significant associations between temperature and antioxidant activities in different fruits. Another study proposed negative correlations between antioxidant activities (FRAP and DPPH essays), and temperature in Pomegranate fruit [111]. Likewise, Wang & Zheng [88] also reported negative impact of MT on the antioxidant properties in the fruits of strawberries. Therefore, it can be inferred from the present findings and previously reported results that temperature may have both positive and negative effects on TPC, TFC and antioxidant activities even in the same part of plant species having different genetic makeup.

##### Impact of Rain Fall and Humidity

Generally, rain fall (Rf), has no substantial connotation with biosynthesis of polyphenols in plants [114]. Correspondingly, in the present study there were no significant correlations of Rf with TPC, TFC, and antioxidant activities in the bulbs and leaves of onion varieties (Tables S1 and S2). In the bulb samples, mostly Rf showed negative correlations with TPC and TFC except in Mustang (V2) and Red Flame (V4) varieties (Table S1). Similarly, in leaves there were negative or weak positive relations between Rf and TPC, except Zeus (V9) variety (Table S2). However, Rf showed negative correlations with TFC in all varieties planted at Kalar Kahar, Lahore and Swabi. These findings were analogous to previous studies [54,113,115] carried out in different food and medicinal plant species. Conversely, significantly positive relationships were noted between Rf and antioxidant activities (Tables S1 and S2). For instance, this was noted with OH radical scavenging activity (+1.000) in the bulbs of Amazon (V8), with molybdate and ferric ion reducing properties at +1.000 and +0.999, respectively (*p* < 0.01 and 0.05) in the leaves of Pulkara (V2), and with ferric ion reduction (+0.998) in the leaves of Red Orb (V2). Strong positive relationships between rainfall and antioxidant activity have also been reported earlier [111,115], specifically with ferric ion and DPPH radicals scavenging potential in medicinal plants. However, Mditshwa et al. [115] reported that antioxidant activity might also be negatively correlated with precipitation, which was consistent with our results as Rf also showed significant (*p* < 0.01) negative associations with H_2_O_2_ radical scavenging activity (−1.000) in the bulbs and with molybdate ion reduction (−0.998) in the leaves of White Pearl (V7). Some studies also reported the interactive effect of temperature and rainfall on TPC and antioxidant activity. Boussaa et al. [111], reported high concentration of TPC in pomegranate fruit during cool and wet season. However, Attanayake et al. [116] mentioned that concentration of TPC decreased in the fruit of Pomegranate in cool and wet regions of Sri Lanka. According to Susanna et al. [117], during high rainfalls and warm temperatures, fruits of Pomegranate had the lowest antioxidant levels, and vice versa in the driest season.

Average level of relative humidity (RH), at three locations showed significantly (*p* < 0.01) negative relations with TFC in the leaves of White Pearl (V7), and TPC in the bulbs of Mustang (V1) as mentioned in Tables S1 and S2. In contrast to our findings, Boussaa et al. [111], reported strong positive correlation between TPC and relative humidity. Likewise, RH depicted strong negative correlations with OH radical scavenging potential in the bulbs and leaves of V4 and V6 varieties. However, significantly (*p* < 0.05) positive relationships were observed for RH with OH and DPPH radicals scavenging activities in the bulbs and leaves of Red Orb (V2), variety (Tables S1 and S2). These findings were in agreement with Boussaa et al. [111], who also reported positive correlations of RH with antioxidant activity as determined by FRAP and DPPH assays.

##### Associations of Surface Pressure, Frost Point and Wind Speed

To the best of our knowledge, impact of surface pressure and dew/frost point on TPC, TFC, and antioxidant activities in plants, specifically in onion has rarely been reported so far. As mentioned in Tables S1 and S2, average levels of surface pressure (SP), at plantation sites of onion varieties had highly significant (*p* < 0.01) positive correlations with TPC and TFC in the bulbs and leaves of Mustang (V1) White Pearl (V7) varieties. Likewise, strong positive correlations were also observed between SP and OH radical scavenging potential in the bulbs of Red Flame (V4) and leaves of Golden Orb (V6). However, SP showed significant (*p* < 0.05) negative associations with OH and DPPH radical scavenging potential in the bulbs and leaves of Red Orb (V2), respectively. On the whole, RH and SP were correlated with DPPH and OH radical scavenging activities and TFC in the leaves of some varieties such as (V2, V6 and V7). Considerable negative correlations of Dew/frost point were calculated with TPC in the leaves and bulbs of V6, and V8, and with molybdate ions reducing potential in the bulbs of Super Sarhad (V3), (Tables S1 and S2).

Relatively, maximum and minimum wind (MWs and MnWs), showed weak and inverse correlations with total phenolic and flavonoid contents in the bulbs and leaves of onion varieties (Tables S1 and S2). These findings were inconsistent with Maslennikov et al. [118], who reported that the pea leaves of the windward side accumulated more phenolic and flavonoid compounds, compared to protected side. Moreover, MWs and MnWs showed considerable positive associations with H_2_O_2_ radical scavenging and molybdate ions reducing activity in the bulbs and leaves of V7 variety. However, there were significant negative correlations with OH radical scavenging capacity (−1.000) in the bulbs of Amazon (V8), and ferric and molybdate ions reducing potential in the leaves of Pulkara (V5). Maslennikov et al. [118], also reported strong associations of wind speed with antioxidant activity, regardless of the method of analysis like DPPH, ABTS, FRAP, in plants.

##### Impact of Altitudinal Variations

Altitudinal variations had strong association with phytochemicals’ contents in plants [58]. Liu et al. [54] reported positive relations between altitude and TFC, while Bernal et al. [119], recorded increase in altitude resulting in decline in the amount of flavonoids in some medicinal plants. Similarly, in our study altitude (Alt.), exhibited highly significant (*p* < 0.01) negative correlations with TFC in the leaves of Amazon (V8), and TPC in the bulbs of Mustang (V1) as mentioned in Tables S1 and S2. But, there were significant positive associations of altitude with OH and DPPH radicals scavenging activities in the bulbs and leaves of Red Orb (V2). These findings were in agreement with Liu et al. [54], and Mpofu et al. [120], who reported positive correlations between altitude and antioxidant activities in wheat *Sinopodophyllum hexandrum*, and *Potentilla fruticosa* etc. Alternatively, we also observed strong negative associations between altitude and OH radical scavenging activity in the bulbs of Red Flame (V4), and leaves of Golden Orb (V6), respectively.

#### 3.4.2. Impact of Edaphic Factors on Total Phenolic, Total Flavonoid Contents and Antioxidant Activities

As far as soil factors are concerned, soil electrical conductivity (EC) was the most prominent factor, which showed significant associations with TPC, TFC and antioxidant potential in the bulbs and leaves of onion varieties (Tables S1 and S2). In the bulb samples, EC depicted noteworthy adverse correlations with DPPH activity in Mustang and Super Sarhad varieties, respectively (Table S1). However, in the leaves of onion varieties, EC exhibited highly significant (*p* < 0.01) positive relationships with TFC and TPC in Golden Orb and Amazon varieties, respectively (Table S2). Likewise, EC was strongly associated with DPPH activity in Amazon, ABTS activity in Red Orb, Golden Orb, and Mustang varieties. These findings were compatible with the fact that EC effected the synthesis of TPC, TFC, and antioxidant activities in plants [121,122,123]. However, it could be vice versa in some cases, as reported by Zargoosh et al. [39], where increased EC did not show any effect on the antioxidant capacity in the fruit of *Scrophularia striata*.

In the present study, soil pH was the second most prominent edaphic factor after EC. In bulb samples (Table S1), soil pH showed substantially positive correlations with OH and DPPH radicals scavenging activities in Golden Orb, and Super Sarhad, respectively. However, pH showed strong negative correlations with TPC, and H_2_O_2_ scavenging activity in Red Flame (V4). In leaves (Table S2), soil pH exhibited substantial positive associations with TPC in Amazon (V8), with OH and H_2_O_2_ activities in Super Sarhad (V3), FRAP values of Red Flame (V4) and H_2_O_2_ radical inhibition potential of White Pearl (V7). Though, soil organic matter (OM), had strong negative correlations with TFC in the bulbs of Mustang (V1) and Pulkara (V5), it showed expressively positive associations with OH and H_2_O_2_ radical scavenging activities (+1.000 and +0.999) in the leaves of White Pearl (V7), and Amazon (V8), varieties respectively, and with molybdate ions reducing potential in the bulbs of Zeus variety (Tables S1 and S2).

Over all, correlation analysis revealed that both climatic and soil factors considerably affected the concentration of TFC, TPC and antioxidant activities in the bulbs and leaves of onion. In addition, disparities in different varieties could be attributed to the complexity of genetic variability. In many cases climatic and edaphic factors showed significant positive relationships with phenolics and antioxidant activities in one variety but were negatively associated in another variety and vice versa. Therefore, it can be concluded that synthesis of secondary metabolites and their bio-activities in various plant parts are mainly influenced by the environmental factors and genetic variation between different species, and different varieties of the same species.

### 3.5. Principal Component Analysis (PCA)

**Figure 8:**
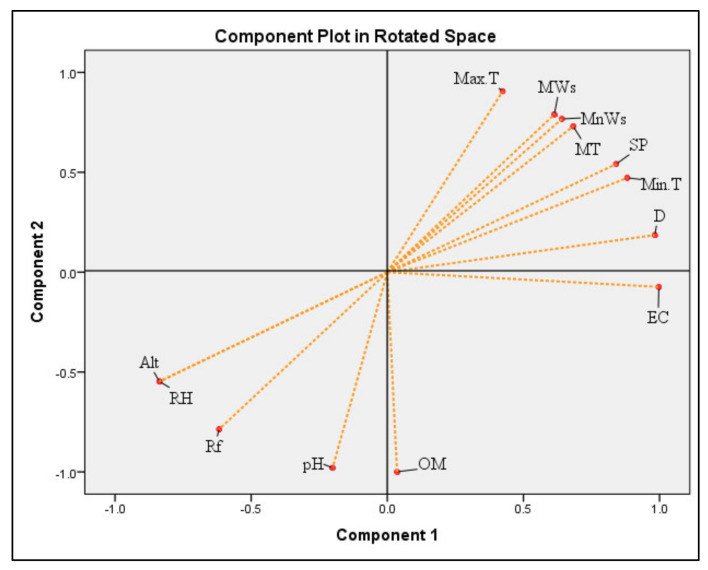
Principal component matrices for climatic and soil factors at planted sites of onion varieties.

Impact of growing conditions on TPC, TFC, and in vitro antioxidant activities in the bulbs and leaves of onion varieties was further evaluated by principal component analysis method. As illustrated in Figure 8, PCA of climatic and edaphic factors of three locations showed two main components. Maximum percentage of cumulative variance was 83.90% in PC1, which indicated positive associations between mean temperature, minimum and maximum wind speed, surface pressure, minimum and maximum temperature and dew or frost point (99.80, 99.40, 98.90, 96.40, 93.20%, and 83.90%, respectively). However, rain fall, altitude, relative humidity and organic matter showed inverse relationships (−0.990, −0.983, −0.98, −0.822 and −0.664, respectively). PC2, revealed weak positive relations between soil electrical conductivity (74.20%), organic matter (74.27%), and pH (57.20%).

Results of PCA between growing conditions (climatic factors and soil properties), TPC, TFC and antioxidant activities in the bulbs and leaves on onion varieties are presented in Table 6. In the bulbs of onion varieties, total variance of PC1 was 70.47%, with maximum loading of MnWs (100%), followed by MWs, MT, Max.T, SP and Min.T (>90%), TPC and FRAP (>80%), and TFC (>50%), whereas, Rf, RH, Alt. and pH showed strong but adverse relations with other variables. Likewise, PC2 indicated that OH and ABTS radicals scavenging properties in the bulbs of onion varieties had strong associations with soil properties i.e., electrical conductivity and organic matter. However, other variables indicated week positive or inverse relations.

Likewise, PCA indicates strong associations of total phenolic, ferric, molybdate and H_2_O_2_ reducing potential in the leaves of onion varieties (PC1), with climatic conditions (rain fall, humidity, and altitude), and soil organic matter. In the leaves of onion varieties, PC1 exhibited significant relations (>90%) of rain fall, and pH with TPC and ferric ion reducing potential. Likewise, RH, Alt., and OM also showed strong associations with FRAP, PMA and H_2_O_2_ (Table 6). However, temperature, wind speed, surface pressure and TFC showed strong negative interactions (Max.T > MWs > MnWs > MT > SP > Min.T > TFC). Results of PC2 showed that electrical conductivity, dew/frost point and surface pressure were more closely associated with ABTS, OH and DPPH radicals’ inhibition capacity in the leaves of onion varieties (Table 6). Our findings of PC analysis revealed strong associations of growing conditions with phytochemicals concentration and their bioactive potential, and also confirmed the results of correlation analysis.

**Figure 9:**
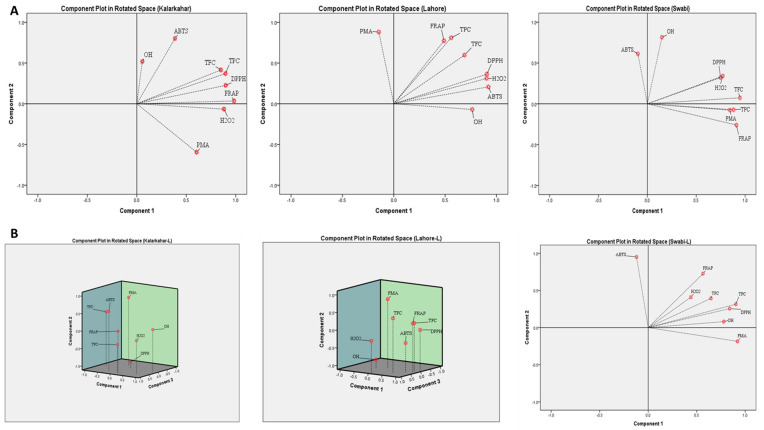
Principal component analysis TPC, TFC and antioxidant properties in bulbs (**A**) and leaves (**B**) of onion varieties planted at diverse locations.

PCA matrices for TPC, TFC, and antioxidant activities in the bulbs of onion varieties harvested from three locations are illustrated in Figure 9A. Total percentage of cumulative variance for PC1 and PC2 was less in Kalar Kahar (77.8%), and Swabi (71.9%), compared to that at Lahore (84.8%). The PC1 for Kalar Kahar indicated strong associations between TPC, TFC, and antioxidant activities with loading values >90%. The PC2 of same location showed strong negative interaction of PMA activity, and weak association of ABTS and OH assays with other variables. PCA analysis indicated that at Lahore, cumulative variance of PC1 was 67.36% (Figure 9A). And in PC1, strong positive relationships were observed between DPPH, H_2_O_2_, TPC, TFC, ABTS and FRAP values, whereas, PMA activity showed diverse behavior, and was placed in PC2 with 81.00% loading value. At Swabi, cumulative variance of PC1 was 55.39% (Figure 9A), depicting significant positive associations between TFC, TPC, PMA, H_2_O_2_ and DPPH with maximum loading percentage. The OH and ABTS radicals’ scavenging activities were placed in PC2 with loading values of (79.9, 61.80%, respectively).

Figure 9B, illustrates results of PCA performed for TPC, TFC and antioxidant activities in the leaves of onion varieties. For onion varieties planted at Swabi, strong positive associations were noted among all variables i.e., TPC, DPPH, FRAP, PMA, TFC, OH and H_2_O_2_ and DPPH (PC1). However, ABTS activity did not show association with other variables, and was placed in PC2. In onion varieties planted at Kalar Kahar (Figure 9B), H_2_O_2_ and DPPH (in PC1), ABTS, FRAP, TPC and TFC (in PC2), and OH and PMA (in PC3), were strongly associated with each other based on their loading values in different components. The PCA of Lahore samples indicated >90% associations between TPC, FRAP and DPPH (PC1), whereas TFC and ABTS also had good positive relations. Likewise, OH and H_2_O_2_ (in PC2), and PMA (in PC3), showed strong relations with each other. Based on the findings of PCA, it can be concluded that phytochemicals have strong associations with bioactive potential of plant species, and these relationships vary in the context of growing environment, and variation in genetic makeup and plant part(s) used.

## 4. Conclusions

Our findings were in agreement with the assumptions that change in the growing environment, and genetic difference had substantial effects on the synthesis, concentration and accumulation of phytochemicals, and their bioactive potential. Significant variations were observed in the concentration of TPC, TFC, and antioxidant potential in the bulbs and leaves of onion varieties. On the whole, concentrations of TPC and TFC, were high in the leaves of onion varieties, compared to the bulbs. Comparatively, bulbs and leaves of Mustang (V1), Red Flame (V4), and Golden Orb (V6) contained more TPC and TFC than other varieties. Likewise, average values of TPC and TFC were high in the bulbs of onion varieties planted at Kalar Kahar, but in the leaves mean concentrations of TPC and TFC were high at Swabi and Kalar Kahar, respectively. Furthermore, mostly free radicals’ scavenging/antioxidant potential was maximum in the bulbs of V1, and leaves of Amazon (V8), varieties. Correlation analysis showed noteworthy associations of climatic and soil factors with total phenolic, total flavonoid contents, and antioxidant potential in the bulbs and leaves of onion. However, PCA revealed complexity of the ecological conditions and genetic makeup that affected the composition and properties of plant species. Findings of the present study could be useful in the selection of best onion varieties enriched in health beneficial secondary metabolites (SM), specifically in the context of diverse growing conditions. However, detailed profiling of phytochemicals, specifically polyphenols using advanced analytical techniques, and in vitro/in vivo bio-activities in onion varieties cultivated under diverse ecological conditions could be more appropriate in order to select best varieties having significant functional properties.

## Figures and Tables

**Table 1:** Total phenolic and flavonoid contents in the bulbs and leaves of onion varieties cultivated at different locations.

Codes	Variety	TPC (mg GAE/100 g, DW)					
		Kalar Kahar (KK)		Lahore (L)		Swabi (S)	
		Bulbs	Leaves	Bulbs	Leaves	Bulbs	Leaves
V1	Mustang	584.3 ± 7.817 ^a^	472.2 ± 19.07 ^abc^	659.5 ± 6.593 ^a^	418.2 ± 10.49 ^d^	478.1 ± 14.25 ^b^	631.1 ± 8.580 ^a^
V2	Red Orb	365.1 ± 11.12 ^c^	474.6 ± 10.15 ^abc^	394.9 ± 2.279 ^b^	469.9 ± 6.363 ^c^	557.2 ± 11.83 ^a^	574.3 ± 11.19 ^b^
V3	Super Sarhad	367.2 ± 3.057 ^c^	486.9 ± 2.402 ^ab^	295.8 ± 2.821 ^e^	368.2 ± 0.697 ^e^	270.7 ± 7.218 ^d^	514.1 ± 11.92 ^c^
V4	Red Flame	315.0 ± 3.750 ^d^	494.1 ± 8.797 ^a^	298.6 ± 8.377 ^de^	370.4 ± 0.701 ^e^	271.4 ± 1.590 ^d^	368.1 ± 2.970 ^d^
V5	Pulkara	276.2 ± 0.985 ^e^	303.0 ± 21.29 ^e^	256.6 ± 16.27 ^f^	364.3 ± 6.126 ^e^	190.3 ± 0.000 ^e^	515.6 ± 16.64 ^c^
V6	Golden Orb	506.7 ± 2.944 ^b^	414.5 ± 9.625 ^d^	355.0 ± 1.115 ^c^	443.6 ± 33.56 ^cd^	328.8 ± 2.492 ^c^	385.7 ± 18.00 ^d^
V7	White Pearl	179.6 ± 1.093 ^g^	417.8 ± 5.671 ^d^	284.3 ± 10.95 ^e^	401.1 ± 11.17 ^de^	255.6 ± 9.869 ^d^	304.8 ± 31.81 ^e^
V8	Amazon	243.8 ± 1.672 ^f^	435.1 ± 29.40 ^cd^	320.0 ± 6.041 ^d^	626.5 ± 2.870 ^a^	269.4 ± 16.55 ^d^	489.5 ± 7.562 ^c^
V9	Zeus	374.6 ± 3.911 ^c^	445.4 ± 4.914 ^bcd^	217.9 ± 9.550 ^g^	530.0 ± 28.19 ^b^	274.6 ± 14.79 ^d^	404.5 ± 19.81 ^d^
Codes	Variety	TFC (mg QE/100 g, DW)					
		Kalar Kahar (KK)		Lahore (L)		Swabi (S)	
		Bulbs	Leaves	Bulbs	Leaves	Bulbs	Leaves
V1	Mustang	303.0 ± 6.670 ^a^	340.2 ± 14.60 ^e^	229.7 ± 9.296 ^a^	318.2 ± 16.77 ^b^	157.7 ± 0.000 ^a^	253.2 ± 21.54 ^ab^
V2	Red Orb	127.9 ± 2.865 ^c^	432.5 ± 10.36 ^ab^	230.3 ± 3.788 ^a^	414.3 ± 19.92 ^a^	145.7 ± 12.83 ^a^	284.5 ± 6.748 ^a^
V3	Super Sarhed	183.6 ± 3.526 ^b^	368.3 ± 2.141 ^e^	93.87 ± 7.764 ^bc^	329.2 ± 10.68 ^b^	99.47 ± 7.289 ^c^	227.1 ± 10.36 ^b^
V4	Red Flame	121.2 ± 4.036 ^c^	479.0 ± 34.05 ^a^	75.80 ± 6.428 ^de^	229.0 ± 12.99 ^c^	98.72 ± 1.361 ^c^	260.7 ± 22.76 ^ab^
V5	Pulkara	101.6 ± 6.442 ^d^	238.6 ± 24.34 ^f^	64.59 ± 1.405 ^e^	202.1 ± 1.129 ^c^	45.30 ± 1.411 ^e^	241.5 ± 16.77 ^ab^
V6	Golden Orb	303.0 ± 2.521 ^a^	352.3 ± 8.099 ^e^	110.8 ± 6.356 ^b^	226.5 ± 23.43 ^c^	122.7 ± 0.000 ^b^	223.8 ± 22.64 ^b^
V7	White Pearl	53.29 ± 1.429 ^f^	393.1 ± 3.037 ^bc^	31.86 ± 1.430 ^f^	138.6 ± 11.48 ^d^	28.95 ± 1.408 ^e^	216.2 ± 5.986 ^b^
V8	Amazon	88.94 ± 2.864 ^e^	375.3 ± 32.49 ^bc^	91.61 ± 8.726 ^cd^	384.6 ± 11.52 ^a^	77.95 ± 0.000 ^d^	232.9 ± 10.44 ^b^
V9	Zeus	120.9 ± 2.457 ^c^	364.4 ± 23.71 ^e^	42.60 ± 1.377 ^f^	316.0 ± 18.27 ^b^	100.1 ± 8.399 ^c^	215.8 ± 14.16 ^b^

Different letters (a–g) showed significant variations in data (*p* < 0.05).

**Table 2:** DPPH and OH radicals scavenging activity in onion varieties.

Bulbs		DPPH (%)			OH (%)		
Codes	Variety	KK	L	S	KK	L	S
V1	Mustang	79.01 ± 1.49 ^a^	61.38 ± 2.09 ^a^	78.75 ± 0.19 ^a^	35.05 ± 0.35 ^f^	64.15 ± 0.84 ^b^	48.41 ± 1.03 ^c^
V2	Red Orb	51.96 ± 1.90 ^c^	48.13 ± 0.38 ^b^	48.47 ± 0.63 ^cd^	45.40 ± 0.18 ^c^	40.87 ± 0.09 ^d^	53.80 ± 0.39 ^b^
V3	Super Sarhad	72.20 ± 1.14 ^b^	41.60 ± 1.3 ^c^	74.38 ± 0.19 ^a^	38.54 ± 0.74 ^e^	42.52 ± 0.29 ^d^	47.18 ± 0.09 ^c^
V4	Red Flame	53.52 ± 0.09 ^c^	38.32 ± 3.72 ^cd^	45.73 ± 0.00 ^d^	62.13 ± 0.11 ^a^	65.50 ± 0.19 ^ab^	56.86 ± 0.29 ^a^
V5	Pulkara	75.30 ± 0.60 ^ab^	41.55 ± 3.50 ^cd^	29.16 ± 1.61 ^e^	41.42 ± 0.09 ^d^	58.76 ± 0.85 ^c^	52.82 ± 0.28 ^b^
V6	Golden Orb	78.05 ± 0.61 ^a^	35.54 ± 2.24 ^d^	53.00 ± 1.99 ^c^	52.94 ± 0.11 ^b^	67.40 ± 0.09 ^a^	41.91 ± 1.12 ^d^
V7	White Pearl	12.35 ± 3.02 ^e^	21.78 ± 0.49 ^e^	23.21 ± 1.53 ^e^	29.53 ± 1.03 ^g^	30.82 ± 0.09 ^f^	36.15 ± 0.37 ^e^
V8	Amazon	41.17 ± 2.41 ^d^	35.90 ± 1.07 ^cd^	61.51 ± 1.04 ^b^	37.93 ± 0.54 ^e^	36.09 ± 1.03 ^e^	56.99 ± 0.18 ^a^
V9	Zeus	45.31 ± 0.97 ^d^	17.08 ± 1.00 ^e^	50.79 ± 6.64 ^cd^	28.86 ± 0.00 ^h^	28.06 ± 0.42 ^g^	31.19 ± 0.85 ^f^
Leaves							
V1	Mustang	60.66 ± 1.50 ^a^	63.74 ± 1.13 ^b^	76.42 ± 0.47 ^a^	51.76 ± 2.23 ^a^	53.83 ± 1.58 ^b^	46.36 ± 3.17 ^ab^
V2	Red Orb	63.85 ± 1.23 ^a^	57.89 ± 0.75 ^cd^	76.20 ± 1.15 ^a^	22.72 ± 3.30 ^c^	24.81 ± 1.16 ^de^	45.11 ± 1.00 ^bc^
V3	Super Sarhad	63.28 ± 2.64 ^a^	50.92 ± 1.33 ^f^	72.02 ± 1.66 ^b^	24.36 ± 2.53 ^c^	30.17 ± 2.46 ^c^	41.00 ± 1.42 ^cd^
V4	Red Flame	56.53 ± 1.33 ^b^	57.43 ± 1.37 ^cd^	63.94 ± 1.50 ^c^	19.91 ± 2.23 ^c^	54.12 ± 0.92 ^b^	28.64 ± 2.41 ^fg^
V5	Pulkara	61.77 ± 0.87 ^a^	52.54 ± 1.33 ^ef^	69.29 ± 0.60 ^b^	24.82 ± 0.00 ^c^	54.89 ± 0.00 ^b^	50.00 ± 0.00 ^a^
V6	Golden Orb	43.55 ± 1.27 ^d^	60.62 ± 2.02 ^bc^	60.41 ± 1.20 ^d^	48.87 ± 0.14 ^a^	61.21 ± 0.76 ^a^	31.61 ± 1.75 ^ef^
V7	White Pearl	45.13 ± 1.37 ^cd^	55.47 ± 1.15 ^de^	51.71 ± 0.41 ^e^	11.63 ± 1.29 ^d^	23.75 ± 0.17 ^e^	33.91 ± 1.49 ^e^
V8	Amazon	54.61 ± 1.16 ^b^	90.69 ± 0.26 ^a^	54.55 ± 1.58 ^e^	32.86 ± 0.14 ^b^	23.75 ± 1.16 ^e^	39.37 ± 0.50 ^d^
V9	Zeus	49.13 ± 0.30 ^c^	88.65 ± 0.58 ^a^	65.50 ± 0.87 ^c^	21.31 ± 1.62 ^c^	28.07 ± 1.63 ^cd^	26.15 ± 0.29 ^g^

KK. Kalar Kahar, L. Lahore, S. Swabi. Different letters (a–g) indicate significant difference between values at *p <* 0.05.

**Table 3:** Hydrogen peroxide and ABTS radicals scavenging activity in onion varieties.

Bulbs		H_2_O_2_ (%)			ABTS (%)		
Codes	Variety	KK	L	S	KK	L	S
V1	Mustang	53.72 ± 1.29 ^a^	55.35 ± 0.93 ^a^	59.76 ± 0.90 ^a^	30.17 ± 2.02 ^a^	65.38 ± 0.99 ^a^	29.88 ± 1.89 ^b^
V2	Red Orb	33.33 ± 0.82 ^e^	51.09 ± 0.54 ^b^	46.43 ± 1.16 ^d^	24.74 ± 1.13 ^ab^	60.66 ± 1.46 ^b^	41.92 ± 0.35 ^a^
V3	Super Sarhad	49.44 ± 1.84 ^b^	48.88 ± 1.11 ^bc^	50.02 ± 1.07 ^c^	19.35 ± 0.82 ^ab^	49.24 ± 0.89 ^d^	19.51 ± 0.86 ^c^
V4	Red Flame	46.44 ± 1.01 ^c^	44.27 ± 0.66 ^d^	41.37 ± 1.28 ^e^	9.240 ± 1.04 ^b^	50.36 ± 0.43 ^cd^	19.88 ± 1.02 ^c^
V5	Pulkara	37.44 ± 1.08 ^b^	41.89 ± 1.42 ^e^	41.89 ± 1.42 ^c^	31.37 ± 12.7 ^a^	49.27 ± 0.57 ^d^	11.03 ± 0.48 ^de^
V6	Golden Orb	41.37 ± 1.28 ^d^	46.43 ± 1.16 ^cd^	53.72 ± 1.29 ^b^	32.80 ± 14.5 ^a^	52.98 ± 1.04 ^c^	16.91 ± 1.00 ^c^
V7	White Pearl	39.14 ± 0.99 ^d^	31.31 ± 0.74 ^h^	26.97 ± 0.87 ^f^	9.221 ± 0.94 ^b^	30.28 ± 1.22 ^e^	11.44 ± 1.12 ^d^
V8	Amazon	33.33 ± 0.82 ^e^	37.44 ± 1.08 ^f^	46.43 ± 1.16 ^d^	10.47 ± 1.89 ^b^	50.20 ± 0.54 ^d^	11.38 ± 1.14 ^d^
V9	Zeus	46.43 ± 1.16 ^bc^	33.33 ± 0.82 ^g^	40.82 ± 0.92 ^e^	11.26 ± 1.59 ^b^	8.560 ± 0.60 ^f^	8.370 ± 0.74 ^e^
Leaves							
V1	Mustang	33.86 ± 1.32 ^b^	42.39 ± 0.47 ^ab^	47.87 ± 1.96 ^b^	10.04 ± 0.98 ^a^	41.36 ± 1.01 ^d^	12.33 ± 0.76 ^b^
V2	Red Orb	39.80 ± 0.75 ^a^	44.18 ± 1.63 ^a^	42.34 ± 0.67 ^c^	11.25 ± 1.36 ^a^	42.89 ± 0.45 ^d^	12.89 ± 0.63 ^b^
V3	Super Sarhad	31.46 ± 0.25 ^b^	33.69 ± 0.41 ^c^	37.13 ± 0.27 ^d^	6.890 ± 0.35 ^a^	37.39 ± 2.77 ^e^	13.46 ± 0.78 ^b^
V4	Red Flame	33.69 ± 0.41 ^b^	40.87 ± 0.35 ^b^	40.87 ± 0.35 ^c^	11.84 ± 2.89 ^a^	56.18 ± 0.67 ^b^	17.58 ± 2.56 ^a^
V5	Pulkara	22.11 ± 2.99 ^c^	32.73 ± 1.88 ^c^	29.23 ± 0.38 ^fg^	7.626 ± 1.77 ^a^	51.17 ± 1.14 ^c^	19.31 ± 0.87 ^a^
V6	Golden Orb	40.87 ± 0.35 ^a^	44.18 ± 1.63 ^a^	31.46 ± 0.25 ^ef^	10.23 ± 1.62 ^a^	51.30 ± 1.24 ^c^	11.59 ± 0.43 ^b^
V7	White Pearl	22.11 ± 2.99 ^c^	24.21 ± 0.70 ^d^	28.45 ± 1.22 ^g^	12.42 ± 8.99 ^a^	19.41 ± 0.77 ^f^	6.062 ± 0.66 ^c^
V8	Amazon	24.21 ± 0.70 ^c^	39.80 ± 0.75 ^b^	51.86 ± 0.43 ^a^	11.53 ± 2.33 ^a^	63.55 ± 1.07 ^a^	19.68 ± 1.15 ^a^
V9	Zeus	31.18 ± 0.85 ^b^	24.21 ± 0.70 ^d^	32.61 ± 0.55 ^e^	11.95 ± 1.21 ^a^	61.58 ± 0.28 ^a^	17.47 ± 0.55 ^a^

KK. Kalar Kahar, L. Lahore, S. Swabi. Different letters (a–g) indicate significant difference between values at *p <* 0.05.

**Table 4:** Ferric and molybdate ions reducing potential in onion varieties.

Bulbs		FRAP (mg GAE/100 g DW)			PMA (mg AAE/100 g DW)		
Codes	Variety	KK	L	S	KK	L	S
V1	Mustang	415.1 ± 10.7 ^a^	373.5 ± 16.5 ^a^	271.2 ± 27.6 ^a^	141.3 ± 9.14 ^ab^	194.2 ± 9.54 ^a^	213.8 ± 0.00 ^a^
V2	Red Orb	153.9 ± 2.56 ^e^	270.4 ± 9.21 ^b^	269.1 ± 13.8 ^a^	85.45 ± 8.35 ^e^	106.2 ± 9.89 ^de^	183.2 ± 17.8 ^b^
V3	Super Sarhad	264.6 ± 4.13 ^c^	278.9 ± 17.2 ^b^	221.1 ± 14.2 ^bc^	112.6 ± 0.62 ^cde^	86.94 ± 1.30 ^ef^	122.2 ± 5.83 ^de^
V4	Red Flame	255.9 ± 5.70 ^c^	182.8 ± 3.87 ^cd^	198.8 ± 9.92 ^bc^	137.0 ± 16.0 ^abc^	91.23 ± 4.58 ^ef^	141.4 ± 14.3 ^cd^
V5	Pulkara	192.2 ± 11.4 ^d^	140.6 ± 2.41 ^e^	137.2 ± 15.5 ^d^	107.1 ± 2.08 ^de^	80.43 ± 2.27 ^f^	72.52 ± 4.55 ^f^
V6	Golden Orb	315.6 ± 5.19 ^b^	203.3 ± 3.75 ^c^	235.5 ± 19.2 ^ab^	157.0 ± 9.43 ^a^	144.7 ± 1.18 ^bc^	133.2 ± 0.00 ^cd^
V7	White Pearl	98.83 ± 3.24 ^f^	194.9 ± 4.95 ^c^	204.0 ± 0.70 ^bc^	136.1 ± 4.16 ^abc^	163.9 ± 15.8 ^b^	140.5 ± 9.55 ^cd^
V8	Amazon	114.6 ± 0.71 ^f^	256.6 ± 13.3 ^b^	189.5 ± 12.8 ^c^	124.8 ± 18.1 ^bcd^	101.8 ± 11.0 ^def^	151.1 ± 8.46 ^c^
V9	Zeus	328.3 ± 23.1 ^b^	152.6 ± 17.9 ^de^	219.8 ± 12.4 ^bc^	160.7 ± 4.03 ^a^	120.3 ± 10.3 ^cd^	99.48 ± 3.59 ^e^
Leaves							
V1	Mustang	74.58 ± 5.98 ^bc^	61.14 ± 10.4 ^e^	132.9 ± 3.90 ^bc^	156.7 ± 9.93 ^e^	179.1 ± 2.67 ^f^	606.8 ± 0.69 ^a^
V2	Red Orb	92.41 ± 0.61 ^a^	91.90 ± 1.98 ^c^	157.7 ± 10.1 ^a^	160.4 ± 12.8 ^e^	460.0 ± 5.28 ^b^	570.7 ± 1.14 ^b^
V3	Super Sarhad	55.12 ± 3.89 ^de^	75.30 ± 0.71 ^de^	128.2 ± 2.52 ^c^	225.3 ± 2.58 ^d^	523.0 ± 0.00 ^a^	623.6 ± 0.72 ^a^
V4	Red Flame	55.89 ± 3.55 ^de^	85.43 ± 4.79 ^cd^	127.8 ± 3.18 ^c^	241.8 ± 5.06 ^d^	385.9 ± 16.3 ^d^	354.0 ± 16.4 ^d^
V5	Pulkara	49.64 ± 4.63 ^e^	37.97 ± 3.81 ^f^	146.0 ± 4.16 ^ab^	332.8 ± 10.9 ^c^	329.6 ± 1.74 ^e^	366.7 ± 9.84 ^cd^
V6	Golden Orb	65.84 ± 4.03 ^cd^	75.49 ± 5.07 ^cde^	120.5 ± 8.09 ^c^	349.3 ± 3.24 ^c^	408.5 ± 0.00 ^cd^	365.6 ± 14.4 ^cd^
V7	White Pearl	63.62 ± 0.00 ^cd^	28.44 ± 3.44 ^f^	71.12 ± 1.82 ^d^	411.6 ± 13.0 ^a^	411.8 ± 0.00 ^cd^	364.7 ± 10.4 ^d^
V8	Amazon	80.62 ± 5.21 ^ab^	184.2 ± 6.75 ^a^	119.9 ± 4.37 ^c^	380.7 ± 0.70 ^b^	424.0 ± 5.23 ^bc^	392.5 ± 0.00 ^c^
V9	Zeus	74.36 ± 5.50 ^bc^	150.2 ± 8.17 ^b^	131.6 ± 4.45 ^bc^	336.9 ± 4.51 ^c^	397.9 ± 33.8 ^cd^	341.9 ± 11.8 ^d^

KK. Kalar Kahar, L. Lahore, S. Swabi. Different letters (a–f) indicate significant difference between values at *p* < 0.05.

**Table 5:** Climatic conditions and soil properties of onion planted sites.

Agro-Climatic Conditions	Codes	Localities		
		Kalar Kahar	Lahore	Swabi
Mean maximum temperature (°C)	Max.T	31.33 ± 9.04	30.85 ± 8.91	28.60 ± 8.26
Mean minimum temperature (°C)	Min.T	17.94 ± 5.18	19.22 ± 5.55	16.43 ± 4.74
Mean temperature (°C)	MT	24.63 ± 7.11	25.03 ± 7.23	22.52 ± 6.50
Average rain fall (mm)	Rf	53.17 ± 15.3	52.35 ± 15.1	64.00 ± 18.5
Relative humidity (%)	RH	41.20 ± 11.9	39.18 ± 11.3	44.46 ± 12.8
Surface pressure (kPa)	SP	96.27 ± 27.8	98.33 ± 28.4	93.03 ± 26.9
Dew/frost point (°C)	D	8.637 ± 2.49	9.546 ± 2.76	8.302 ± 2.40
Mean maximum wind speed (m/s)	MWs	3.423 ± 0.99	3.457 ± 1.00	2.842 ± 0.82
Mean minimum wind speed (m/s)	MnWs	1.004 ± 0.29	1.045 ± 0.30	0.653 ± 0.19
Altitude (m)	Alt.	401.6	213.0	706.0
pH	pH	6.085 ± 0.03	6.195 ± 0.14	6.360 ± 0.05
Electrical conductivity (dSm^−1^)	EC	2.135 ± 0.36	12.77 ± 0.77	1.960 ± 0.73
Soil organic matter (%)	Om	0.875 ± 0.03	0.900 ± 0.07	0.925 ± 0.03

Source: Pakistan metrological department (PMD).

**Table 6:** Principal component matrices for mean values of climatic conditions, TPC, TFC and antioxidant activities in onion.

Variables	Bulbs		Leaves	
	PC1	PC2	PC1	PC2
Eigen value	14.79	6.201	14.71	6.290
Total variance (%)	70.47	29.57	70.04	29.95
Cumulative variance (%)	70.47	100.0	70.04	100.0
Total phenolic content (TPC)	0.869	−0.494	0.994	0.106
Total flavonoid content (TFC)	0.562	−0.827	−0.730	−0.684
FRAP assay	0.851	−0.525	0.967	0.256
PMA assay	−0.940	−0.341	0.809	0.588
DPPH assay	−0.356	−0.935	0.665	0.747
OH assay	−0.292	0.957	0.468	0.884
H_2_O_2_ assay	−0.815	0.580	0.749	0.662
ABTS assay	0.609	0.793	−0.334	0.943
Maximum temperature (Max.T)	0.966	−0.257	−0.997	−0.082
Minimum temperature (Min.T)	0.929	0.371	−0.848	0.530
Mean temperature (MT)	0.999	0.052	−0.974	0.228
Rain fall (Rf)	−0.999	0.033	0.990	−0.144
Relative humidity (RH)	−0.958	−0.288	0.892	−0.453
Surface pressure (SP)	0.956	0.295	−0.888	0.459
Dew/frost point (D)	0.773	0.635	−0.648	0.761
Maximum wind speed (MWs)	0.999	−0.042	−0.991	0.135
Minimum wind speed (MnWs)	1.000	−0.011	−0.986	0.166
pH	−0.878	0.480	0.948	0.317
Electrical conductivity (EC)	0.585	0.811	−0.432	0.902
Organic matter (OM)	−0.791	0.611	0.887	0.462
Altitude (Alt.)	−0.957	−0.289	0.891	−0.454

## Data Availability

Not applicable.

## Supplementary Materials

The following supporting information can be downloaded at: https://www.mdpi.com/article/10.3390/plants11070950/s1. Table S1: Correlations of total phenolic, total flavonoid contents and antioxidant activities in onion bulbs with growing conditions. Table S2: Correlations of total phenolic, flavonoid contents, and antioxidant activities in onion leaves with growing conditions. Figure S1: Correlation analysis between TPC, TFC and antioxidant activities in the bulbs of onion varieties. Figure S2: Correlation analysis between TPC, TFC and antioxidant activities in the leaves of onion varieties.
