# Supplementary material for: Variations in Total Phenolic, Total Flavonoid Contents, and Free Radicals’ Scavenging Potential of Onion Varieties Planted under Diverse Environmental Conditions
Source: Plants (Basel). 2022 Mar 31;11(7):950. doi: 10.3390/plants11070950 (PMC9002954; doi:10.3390/plants11070950)
Supplement: Supplementary file 1 [file plants-11-00950-s001.zip › plants-1628620-supplementary.pdf]

plants

MDPI

Table S1. Correlations of total phenolic, total flavonoid contents and antioxidant activities in onion bulbs with growing conditions

**V1-B**	Max.T	Min.T	MT	Rf	RH	SP	D	MWs	MnWs	pH	EC	Om	Alt.
TPC	0.831	.999(*)	0.962	-0.935	-.999(*)	1.000(*)	0.937	0.933	0.946	-0.675	0.816	-0.551	-.999(*)
TFC	0.935	0.536	0.779	-0.830	-0.608	0.602	0.258	0.833	0.813	-0.992	0.000	-.999(*)	-0.608
FRAP	0.993	0.724	0.908	-0.940	-0.782	0.777	0.484	0.942	0.929	-0.993	0.242	-0.962	-0.782
PMA	-0.815	-0.307	-0.596	0.663	0.388	-0.382	-0.007	-0.667	-0.640	0.930	0.251	0.976	0.389
DPPH	-0.338	-0.834	-0.613	0.543	0.783	-0.787	-0.961	-0.538	-0.568	0.105	-1.000(*)	-0.052	0.783
OH	-0.118	0.500	0.195	-0.110	-0.422	0.429	0.737	0.105	0.140	0.350	0.886	0.492	-0.422
H_2O_2	-0.995	-0.739	-0.917	0.947	0.795	-0.791	-0.503	-0.949	-0.937	0.990	-0.264	0.955	0.795
ABTS	0.695	0.987	0.884	-0.840	-0.970	0.971	0.990	0.837	0.856	-0.505	0.919	-0.364	-0.970
**V2-B**	Max.T	Min.T	MT	Rf	RH	SP	D	MWs	MnWs	pH	EC	Om	Alt.
TPC	-1.000(*)	-0.813	-0.957	0.979	0.861	-0.858	-0.601	-0.980	-0.972	0.966	-0.375	0.914	0.861
TFC	0.194	0.742	0.487	-0.411	-0.681	0.686	0.909	0.406	0.438	0.045	0.986	0.201	-0.681
FRAP	-0.628	-0.037	-0.357	0.435	0.124	-0.118	0.264	-0.440	-0.408	0.795	0.504	0.880	0.124
PMA	-.999(*)	-0.778	-0.939	0.965	0.830	-0.826	-0.553	-0.966	-0.956	0.980	-0.320	0.936	0.830
DPPH	0.571	-0.034	0.289	-0.370	-0.053	0.046	-0.333	0.375	0.342	-0.749	-0.564	-0.844	-0.053
OH	-0.869	-0.992	-0.979	0.958	.999(*)	-.999(*)	-0.910	-0.957	-0.967	0.726	-0.772	0.610	.999(*)
H_2O_2	-0.419	0.207	-0.118	0.203	-0.121	0.128	0.491	-0.208	-0.173	0.623	0.699	0.738	-0.121
ABTS	0.223	0.762	0.514	-0.438	-0.703	0.708	0.921	0.434	0.465	0.015	0.990	0.171	-0.703
**V3-B**	Max.T	Min.T	MT	Rf	RH	SP	D	MWs	MnWs	pH	EC	Om	Alt.
TPC	0.809	0.296	0.588	-0.655	-0.378	0.372	-0.004	0.659	0.632	-0.925	-0.262	-0.973	-0.378
TFC	0.592	-0.008	0.314	-0.394	-0.079	0.072	-0.308	0.399	0.366	-0.766	-0.543	-0.857	-0.079
FRAP	0.919	0.972	0.996	-0.984	-0.989	0.988	0.858	0.983	0.989	-0.798	0.696	-0.695	-0.989
PMA	-0.584	-0.954	-0.807	0.753	0.924	-0.927	-1.000(**)	-0.75	-0.773	0.375	-0.966	0.225	0.924
DPPH	-0.406	-0.872	-0.668	0.602	0.826	-0.83	-0.979	-0.598	-0.626	0.177	-.999(*)	0.021	0.826
OH	-0.951	-0.578	-0.809	0.857	0.647	-0.641	-0.306	-0.859	-0.841	.997(*)	-0.051	0.997	0.647

H_2O_2	-0.777	-.999(*)	-0.933	0.899	0.992	-0.993	-0.965	-0.897	-0.912	0.605	-0.865	0.473	0.992
ABTS	-0.437	0.188	-0.137	0.221	-0.102	0.109	0.475	-0.227	-0.192	0.638	0.685	0.75	-0.102
**V4-B**	Max.T	Min.T	MT	Rf	RH	SP	D	MWs	MnWs	pH	EC	Om	Alt.
TPC	0.977	0.654	0.862	-0.902	-0.717	0.712	0.396	0.905	0.889	-1.000(*)	0.146	-0.984	-0.717
TFC	0.159	-0.463	-0.154	0.069	0.384	-0.391	-0.708	-0.063	-0.099	-0.389	-0.866	-0.528	0.384
FRAP	0.461	-0.161	0.164	-0.248	0.075	-0.082	-0.450	0.253	0.219	-0.659	-0.665	-0.768	0.075
PMA	-0.422	-0.881	-0.682	0.617	0.836	-0.840	-0.982	-0.612	-0.640	0.195	-.997(*)	0.039	0.836
DPPH	0.178	-0.446	-0.134	0.049	0.366	-0.373	-0.694	-0.044	-0.079	-0.407	-0.856	-0.545	0.366
OH	0.846	0.997	0.969	-0.945	-1.000(**)	1.000(**)	0.927	0.943	0.954	-0.695	0.800	-0.574	-1.000(**)
H_2O_2	0.962	0.608	0.831	-0.876	-0.675	0.670	0.342	0.878	0.861	-.999(*)	0.088	-0.993	-0.675
ABTS	0.116	0.687	0.417	-0.338	-0.621	0.626	0.873	0.333	0.366	0.124	0.969	0.277	-0.621
**V5-B**	Max.T	Min.T	MT	Rf	RH	SP	D	MWs	MnWs	pH	EC	Om	Alt.
TPC	.998(*)	0.767	0.933	-0.960	-0.820	0.816	0.539	0.962	0.951	-0.984	0.304	-0.914	-0.820
TFC	0.859	0.381	0.658	-0.720	-0.460	0.454	0.086	0.724	0.699	-0.954	-0.173	-.998(*)	-0.461
FRAP	0.677	0.102	0.416	-0.493	-0.188	0.182	-0.201	0.497	0.466	-0.83	-0.447	-0.938	-0.188
PMA	0.789	0.265	0.561	-0.630	-0.348	0.341	-0.037	0.634	0.606	-0.911	-0.293	-0.982	-0.348
DPPH	.998(*)	0.761	0.929	-0.958	-0.815	0.811	0.532	0.959	0.948	-0.985	0.295	-0.917	-0.815
OH	-0.338	0.292	-0.031	0.116	-0.208	0.214	0.566	-0.122	-0.086	0.548	0.759	0.730	-0.208
H_2O_2	-0.636	-0.048	-0.366	0.444	0.134	-0.127	0.255	-0.449	-0.417	0.798	0.495	0.918	0.134
ABTS	0.686	0.985	0.877	-0.833	-0.966	0.968	0.991	0.830	0.850	-0.498	0.924	-0.279	-0.966
**V6-B**	Max.T	Min.T	MT	Rf	RH	SP	D	MWs	MnWs	pH	EC	Om	Alt.
TPC	0.735	0.183	0.490	-0.562	-0.268	0.262	-0.120	0.567	0.537	-0.873	-0.372	-0.963	-0.268
TFC	0.592	-0.008	0.314	-0.394	-0.079	0.072	-0.308	0.399	0.366	-0.764	-0.542	-0.894	-0.079
FRAP	0.396	-0.232	0.093	-0.178	0.146	-0.153	-0.513	0.183	0.148	-0.599	-0.717	-0.771	0.146
PMA	0.930	0.525	0.771	-0.823	-0.597	0.592	0.245	0.826	0.805	-0.990	-0.013	-0.995	-0.597
DPPH	0.265	-0.365	-0.046	-0.039	0.282	-0.289	-0.628	0.045	0.009	-0.482	-0.807	-0.675	0.282
OH	0.720	0.992	0.899	-0.859	-0.978	0.979	0.984	0.856	0.874	-0.539	0.905	-0.325	-0.978
H_2O_2	-0.968	-0.625	-0.843	0.886	0.691	-0.686	-0.362	-0.888	-0.871	1.000(*)	-0.110	0.976	0.691

ABTS	0.786	1.000(*)	0.938	-0.905	-0.994	0.994	0.961	0.903	0.918	-0.620	0.858	-0.417	-0.994
**V7-B**	Max.T	Min.T	MT	Rf	RH	SP	D	MWs	MnWs	pH	EC	Om	Alt.
TPC	-0.409	0.219	-0.107	0.191	-0.133	0.140	0.502	-0.197	-0.162	0.610	0.707	0.780	-0.133
TFC	0.716	0.157	0.466	-0.540	-0.242	0.235	-0.147	0.544	0.514	-0.859	-0.397	-0.956	-0.242
FRAP	-0.695	-0.126	-0.438	0.514	0.212	-0.205	0.178	-0.518	-0.487	0.843	0.425	0.946	0.212
PMA	0.206	0.750	0.498	-0.422	-0.690	0.695	0.914	0.417	0.449	0.028	0.988	0.263	-0.690
DPPH	-0.725	-0.169	-0.477	0.550	0.254	-0.247	0.135	-0.555	-0.525	0.865	0.386	0.959	0.254
OH	-1.000(*)	-0.790	-0.945	0.970	0.840	-0.836	-0.569	-0.971	-0.962	0.977	-0.338	0.899	0.840
H_2O_2	0.980	0.904	0.994	-1.000(*)	-0.938	0.936	0.734	1.000(*)	.998(*)	-0.907	0.535	-0.782	-0.938
ABTS	-0.011	0.590	0.299	-0.216	-0.517	0.523	0.805	0.211	0.245	0.243	0.931	0.465	-0.517
**V8-B**	Max.T	Min.T	MT	Rf	RH	SP	D	MWs	MnWs	pH	EC	Om	Alt.
TPC	-0.635	-0.971	-0.842	0.793	0.946	-0.949	-.998(*)	-0.790	-0.811	0.437	-0.948	0.213	0.946
TFC	0.410	0.874	0.672	-0.606	-0.829	0.832	0.980	0.601	0.630	-0.186	.998(*)	0.051	-0.828
FRAP	-0.196	0.430	0.117	-0.031	-0.349	0.356	0.681	0.026	0.061	0.419	0.847	0.621	-0.349
PMA	-0.797	-1.000(**)	-0.945	0.913	0.996	-0.996	-0.956	-0.911	-0.925	0.634	-0.848	0.434	0.996
DPPH	-0.935	-0.961	-.999(*)	0.991	0.981	-0.980	-0.834	-0.990	-0.995	0.827	-0.663	0.671	0.981
OH	-0.970	-0.922	-.998(*)	1.000(*)	0.953	-0.950	-0.764	-1.000(*)	-1.000(**)	0.887	-0.572	0.753	0.953
H_2O_2	-0.989	-0.705	-0.896	0.930	0.764	-0.760	-0.460	-0.932	-0.919	0.996	-0.216	0.947	0.764
ABTS	0.330	0.829	0.606	-0.536	-0.778	0.782	0.959	0.531	0.561	-0.101	1.000(*)	0.136	-0.778
**V9-B**	Max.T	Min.T	MT	Rf	RH	SP	D	MWs	MnWs	pH	EC	Om	Alt.
TPC	0.296	-0.335	-0.014	-0.072	0.251	-0.258	-0.602	0.077	0.041	-0.510	-0.788	-0.699	0.251
TFC	0.117	0.688	0.419	-0.339	-0.623	0.628	0.874	0.334	0.368	0.117	0.970	0.348	-0.623
FRAP	0.296	-0.334	-0.014	-0.072	0.251	-0.258	-0.602	0.077	0.042	-0.510	-0.787	-0.699	0.251
PMA	0.858	0.379	0.657	-0.719	-0.458	0.452	0.084	0.723	0.697	-0.954	-0.176	-.998(*)	-0.459
DPPH	0.074	-0.537	-0.237	0.153	0.462	-0.468	-0.766	-0.148	-0.183	-0.305	-0.905	-0.521	0.462
OH	-0.916	-0.974	-0.995	0.983	0.990	-0.989	-0.861	-0.982	-0.988	0.797	-0.701	0.632	0.990
H_2O_2	0.083	-0.530	-0.229	0.145	0.454	-0.460	-0.760	-0.140	-0.175	-0.313	-0.902	-0.528	0.454

ABTS	-0.386	-0.861	-0.652	0.585	0.814	-0.818	-0.974	-0.580	-0.609	0.160	-.999(*)	-0.077	0.814

Table S2. Correlations of total phenolic, flavonoid contents, and antioxidant activities in onion leaves with growing conditions

**V1-L**	Max.T	Min.T	MT	Rf	RH	SP	D	MWs	MnWs	pH	EC	Om	Alt.
TPC	0.061	-0.549	-0.251	0.167	0.474	-0.480	-0.774	-0.162	-0.197	-0.296	-0.911	-0.442	0.474
TFC	0.497	0.917	0.741	-0.680	-0.879	0.882	0.995	0.677	0.702	-0.276	0.988	-0.123	-0.879
FRAP	-0.942	-0.956	-1.000(*)	0.994	0.978	-0.976	-0.823	-0.993	-0.997	0.835	-0.649	0.739	0.978
PMA	-0.993	-0.868	-0.981	0.994	0.908	-0.905	-0.679	-0.995	-0.991	0.935	-0.466	0.869	0.908
DPPH	-1.000(*)	-0.789	-0.945	0.969	0.839	-0.836	-0.568	-0.970	-0.961	0.976	-0.337	0.930	0.839
OH	0.906	0.979	0.992	-0.978	-0.993	0.992	0.873	0.977	0.984	-0.779	0.718	-0.672	-0.993
H_2O_2	-0.886	-0.432	-0.699	0.758	0.509	-0.503	-0.141	-0.761	-0.737	0.971	0.119	0.996	0.509
ABTS	0.288	0.804	0.570	-0.498	-0.749	0.754	0.945	0.493	0.524	-0.053	.997(*)	0.104	-0.749
**V2-L**	Max.T	Min.T	MT	Rf	RH	SP	D	MWs	MnWs	pH	EC	Om	Alt.
TPC	-0.253	-0.782	-0.54	0.466	0.725	-0.729	-0.933	-0.461	-0.492	0.016	-0.994	-0.140	0.725
TFC	0.732	0.994	0.907	-0.868	-0.981	0.982	0.981	0.865	0.882	-0.549	0.897	-0.412	-0.981
FRAP	-0.985	-0.892	-0.990	.998(*)	0.928	-0.925	-0.715	-.999(*)	-0.996	0.916	-0.511	0.842	0.928
PMA	-0.815	-0.306	-0.596	0.663	0.388	-0.382	-0.006	-0.666	-0.640	0.929	0.252	0.976	0.388
DPPH	-0.882	-0.989	-0.985	0.966	.998(*)	-.997(*)	-0.898	-0.965	-0.973	0.745	-0.754	0.632	.998(*)
OH	-0.997	-0.847	-0.973	0.989	0.890	-0.887	-0.648	-0.99	-0.984	0.949	-0.43	0.888	0.89
H_2O_2	-0.254	0.376	0.058	0.028	-0.294	0.300	0.637	-0.033	0.002	0.476	0.814	0.608	-0.294
ABTS	0.307	0.816	0.586	-0.515	-0.762	0.767	0.952	0.51	0.541	-0.072	.999(*)	0.084	-0.762
**V3-L**	Max.T	Min.T	MT	Rf	RH	SP	D	MWs	MnWs	pH	EC	Om	Alt.
TPC	0.636	0.048	0.367	-0.445	-0.135	0.128	-0.254	0.450	0.418	-0.801	-0.495	-0.885	-0.135
TFC	0.657	0.977	0.858	-0.811	-0.955	0.957	0.996	0.808	0.828	-0.459	0.938	-0.315	-0.955
FRAP	-0.994	-0.734	-0.913	0.945	0.790	-0.786	-0.496	-0.947	-0.934	0.991	-0.256	0.958	0.790
PMA	-0.804	-0.289	-0.581	0.649	0.371	-0.365	0.012	-0.653	-0.625	0.922	0.269	0.971	0.371
DPPH	-0.706	-0.989	-0.890	0.848	0.973	-0.974	-0.987	-0.845	-0.863	0.517	-0.913	0.377	0.973
OH	-0.983	-0.677	-0.877	0.915	0.738	-0.734	-0.424	-0.917	-0.903	.999(*)	-0.177	0.978	0.738
H_2O_2	-0.972	-0.638	-0.852	0.894	0.703	-0.698	-0.378	-0.896	-0.880	1.000(**)	-0.126	0.987	0.703

ABTS	0.151	0.713	0.450	-0.371	-0.649	0.654	0.891	0.367	0.399	0.088	0.978	0.242	-0.649
**V4-L**	Max.T	Min.T	MT	Rf	RH	SP	D	MWs	MnWs	pH	EC	Om	Alt.
TPC	.999(*)	0.832	0.966	-0.985	-0.877	0.874	0.627	0.986	0.979	-0.957	0.405	-0.900	-0.877
TFC	0.139	0.704	0.438	-0.359	-0.639	0.644	0.885	0.354	0.387	0.101	0.975	0.255	-0.639
FRAP	-0.968	-0.624	-0.842	0.885	0.690	-0.685	-0.360	-0.888	-0.871	1.000(**)	-0.108	0.990	0.690
PMA	-0.459	0.164	-0.162	0.246	-0.077	0.084	0.453	-0.251	-0.216	0.657	0.667	0.767	-0.077
DPPH	-.999(*)	-0.832	-0.966	0.985	0.878	-0.874	-0.628	-0.986	-0.979	0.957	-0.406	0.900	0.878
OH	0.11	0.683	0.412	-0.332	-0.616	0.622	0.871	0.327	0.36	0.130	0.968	0.283	-0.616
H_2O_2	-0.636	-0.048	-0.366	0.444	0.134	-0.127	0.255	-0.449	-0.417	0.801	0.495	0.885	0.134
ABTS	0.237	0.771	0.525	-0.451	-0.713	0.717	0.927	0.446	0.477	0.001	0.992	0.157	-0.713
**V5-L**	Max.T	Min.T	MT	Rf	RH	SP	D	MWs	MnWs	pH	EC	Om	Alt.
TPC	-0.110	0.507	0.203	-0.118	-0.430	0.436	0.742	0.113	0.148	0.343	0.890	0.485	-0.430
TFC	0.240	0.773	0.528	-0.454	-0.715	0.720	0.928	0.449	0.481	-0.003	0.993	0.153	-0.715
FRAP	-0.965	-0.930	-.999(*)	.999(*)	0.958	-0.956	-0.776	-.999(*)	-1.000(**)	0.876	-0.587	0.790	0.958
PMA	-0.970	-0.922	-.997(*)	1.000(**)	0.952	-0.950	-0.763	-1.000(*)	-1.000(**)	0.885	-0.571	0.802	0.952
DPPH	-0.733	-0.994	-0.907	0.868	0.981	-0.983	-0.980	-0.865	-0.883	0.550	-0.897	0.413	0.981
OH	-0.512	0.104	-0.221	0.304	-0.017	0.024	0.398	-0.309	-0.275	0.701	0.621	0.804	-0.017
H_2O_2	-0.352	0.278	-0.046	0.131	-0.193	0.200	0.553	-0.136	-0.101	0.565	0.749	0.687	-0.193
ABTS	0.096	0.672	0.399	-0.319	-0.605	0.611	0.864	0.314	0.347	0.143	0.964	0.296	-0.605
**V6-L**	Max.T	Min.T	MT	Rf	RH	SP	D	MWs	MnWs	pH	EC	Om	Alt.
TPC	-0.625	-0.968	-0.836	0.786	0.942	-0.945	-.999(*)	-0.783	-0.804	0.422	-0.952	0.275	0.942
TFC	0.364	0.849	0.634	-0.566	-0.800	0.804	0.969	0.561	0.590	-0.132	1.000(**)	0.024	-0.800
FRAP	-1.000(**)	-0.801	-0.951	0.974	0.850	-0.846	-0.584	-0.975	-0.967	0.972	-0.355	0.923	0.850
PMA	0.088	0.667	0.392	-0.312	-0.599	0.605	0.860	0.307	0.340	0.151	0.962	0.303	-0.599
DPPH	-0.627	-0.037	-0.356	0.434	0.123	-0.117	0.265	-0.439	-0.407	0.794	0.505	0.879	0.123
OH	0.829	.999(*)	0.961	-0.934	-.999(*)	.999(*)	0.938	0.932	0.945	-0.672	0.818	-0.549	-.999(*)
H_2O_2	0.913	0.975	0.995	-0.982	-0.991	0.990	0.864	0.981	0.987	-0.791	0.705	-0.685	-0.991

ABTS	0.323	0.825	0.600	-0.529	-0.773	0.777	0.957	0.525	0.554	-0.089	.999(*)	0.068	-0.773
**V7-L**	Max.T	Min.T	MT	Rf	RH	SP	D	MWs	MnWs	pH	EC	Om	Alt.
TPC	0.423	0.881	0.682	-0.617	-0.837	0.840	0.982	0.613	0.641	-0.196	.997(*)	-0.040	-0.837
TFC	-0.127	0.492	0.186	-0.101	-0.414	0.420	0.731	0.096	0.131	0.359	0.882	0.500	-0.414
FRAP	-0.500	-0.919	-0.743	0.683	0.881	-0.884	-0.995	-0.679	-0.705	0.280	-0.987	0.127	0.881
PMA	0.986	0.891	0.990	-.998(*)	-0.927	0.925	0.714	.999(*)	0.996	-0.917	0.509	-0.844	-0.927
DPPH	-0.317	0.314	-0.008	0.093	-0.230	0.237	0.585	-0.099	-0.063	0.533	0.774	0.658	-0.230
OH	-0.917	-0.497	-0.750	0.803	0.571	-0.565	-0.213	-0.807	-0.785	0.986	0.045	1.000(**)	0.571
H_2O_2	-0.986	-0.691	-0.887	0.923	0.751	-0.747	-0.442	-0.925	-0.911	.997(*)	-0.196	0.974	0.751
ABTS	0.754	.997(*)	0.920	-0.883	-0.987	0.988	0.974	0.881	0.897	-0.577	0.882	-0.442	-0.987
**V8-L**	Max.T	Min.T	MT	Rf	RH	SP	D	MWs	MnWs	pH	EC	Om	Alt.
TPC	-0.975	-0.647	-0.858	0.899	0.711	-0.707	-0.389	-0.901	-0.885	1.000(**)	-0.138	0.985	0.711
TFC	0.834	.998(*)	0.964	-0.937	-1.000(*)	1.000(*)	0.935	0.936	0.948	-0.679	0.812	-0.556	-1.000(*)
FRAP	-0.026	0.577	0.284	-0.201	-0.504	0.510	0.796	0.196	0.230	0.263	0.925	0.411	-0.504
PMA	0.090	0.668	0.393	-0.313	-0.600	0.606	0.860	0.308	0.342	0.150	0.963	0.302	-0.600
DPPH	0.352	0.842	0.624	-0.555	-0.792	0.796	0.965	0.550	0.580	-0.119	1.000(**)	0.037	-0.792
OH	-0.707	-0.990	-0.891	0.849	0.973	-0.975	-0.987	-0.846	-0.865	0.519	-0.912	0.379	0.973
H_2O_2	-0.908	-0.477	-0.735	0.790	0.552	-0.546	-0.192	-0.793	-0.771	0.982	0.068	.999(*)	0.552
ABTS	0.210	0.754	0.502	-0.426	-0.693	0.698	0.916	0.422	0.454	0.028	0.989	0.184	-0.693
**V9-L**	Max.T	Min.T	MT	Rf	RH	SP	D	MWs	MnWs	pH	EC	Om	Alt.
TPC	-.999(*)	-0.770	-0.934	0.961	0.823	-0.819	-0.543	-0.963	-0.953	0.982	-0.308	0.941	0.823
TFC	0.794	1.000(**)	0.943	-0.911	-0.995	0.996	0.957	0.909	0.923	-0.627	0.851	-0.498	-0.995
FRAP	-0.437	0.188	-0.137	0.222	-0.102	0.109	0.474	-0.227	-0.192	0.638	0.685	0.751	-0.102
PMA	0.280	0.799	0.563	-0.490	-0.743	0.748	0.943	0.486	0.516	-0.044	0.997	0.113	-0.743
DPPH	-0.067	0.544	0.245	-0.161	-0.468	0.475	0.771	0.156	0.191	0.302	0.909	0.447	-0.468
OH	-0.399	0.229	-0.096	0.181	-0.144	0.150	0.511	-0.186	-0.151	0.605	0.715	0.722	-0.144
H_2O_2	-0.495	-0.917	-0.740	0.679	0.878	-0.882	-0.994	-0.675	-0.701	0.275	-0.988	0.121	0.878

ABTS	0.254	0.782	0.540	-0.466	-0.725	0.730	0.933	0.462	0.493	-0.017	0.994	0.140	-0.725

Max.T. Mean maximum temperature, Min.T. Mean minimum temperature, MT. Mean temperature, Rf. Average rain fall, RH. Relative humidity, SP. Surface pressure, D. Dew/frost point, MWs. Maximum wind speed, MnWs. Minimum wind speed, EC. Electrical conductivity, Om. Organic matter, Alt. altitude. *Correlation is significant at the 0.05 level (2-tailed)., **Correlation is significant at the 0.01 level (2-tailed).

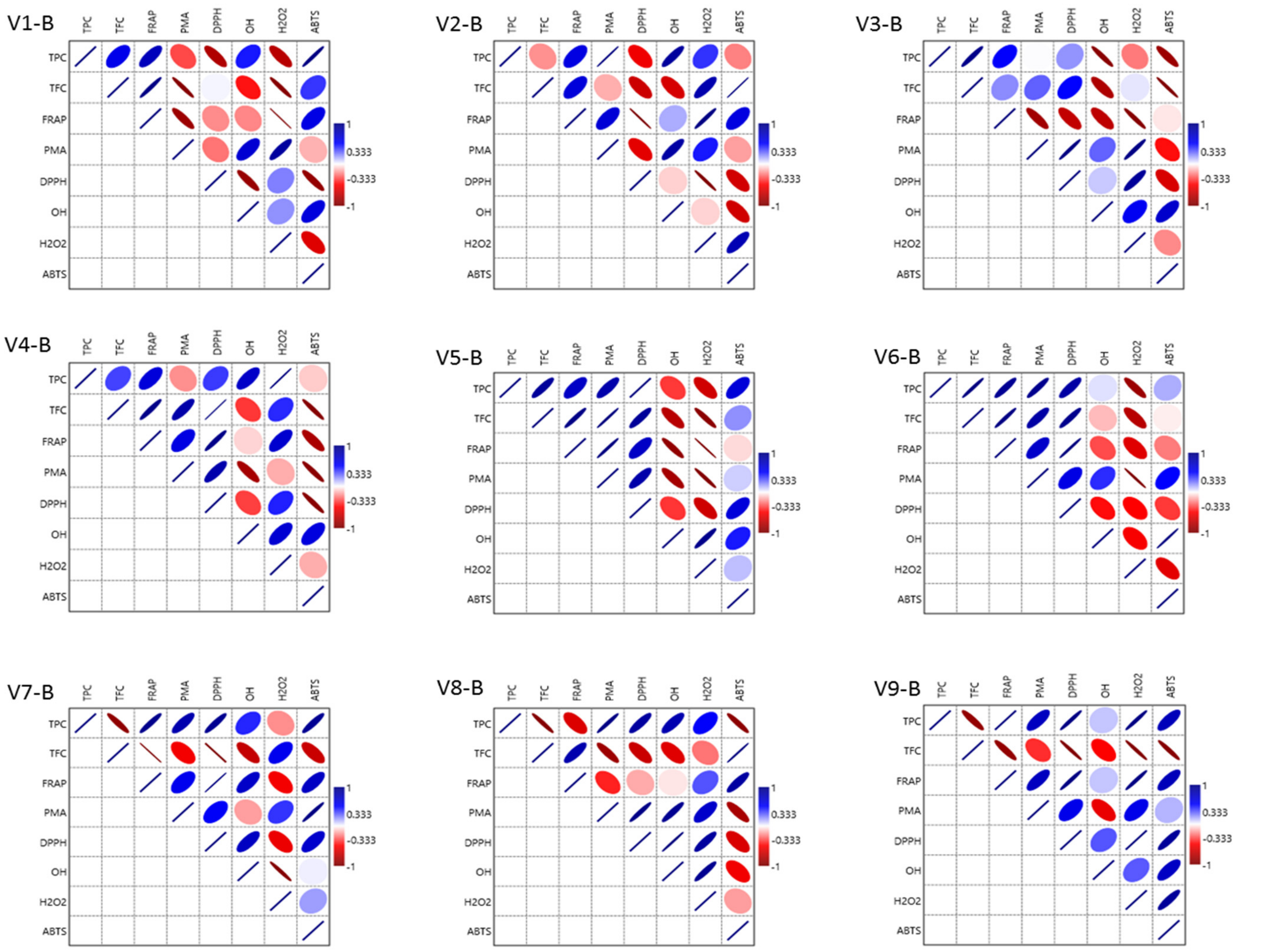

Figure S1. Correlation analysis between TPC, TFC and antioxidant activities in the bulbs of onion varieties

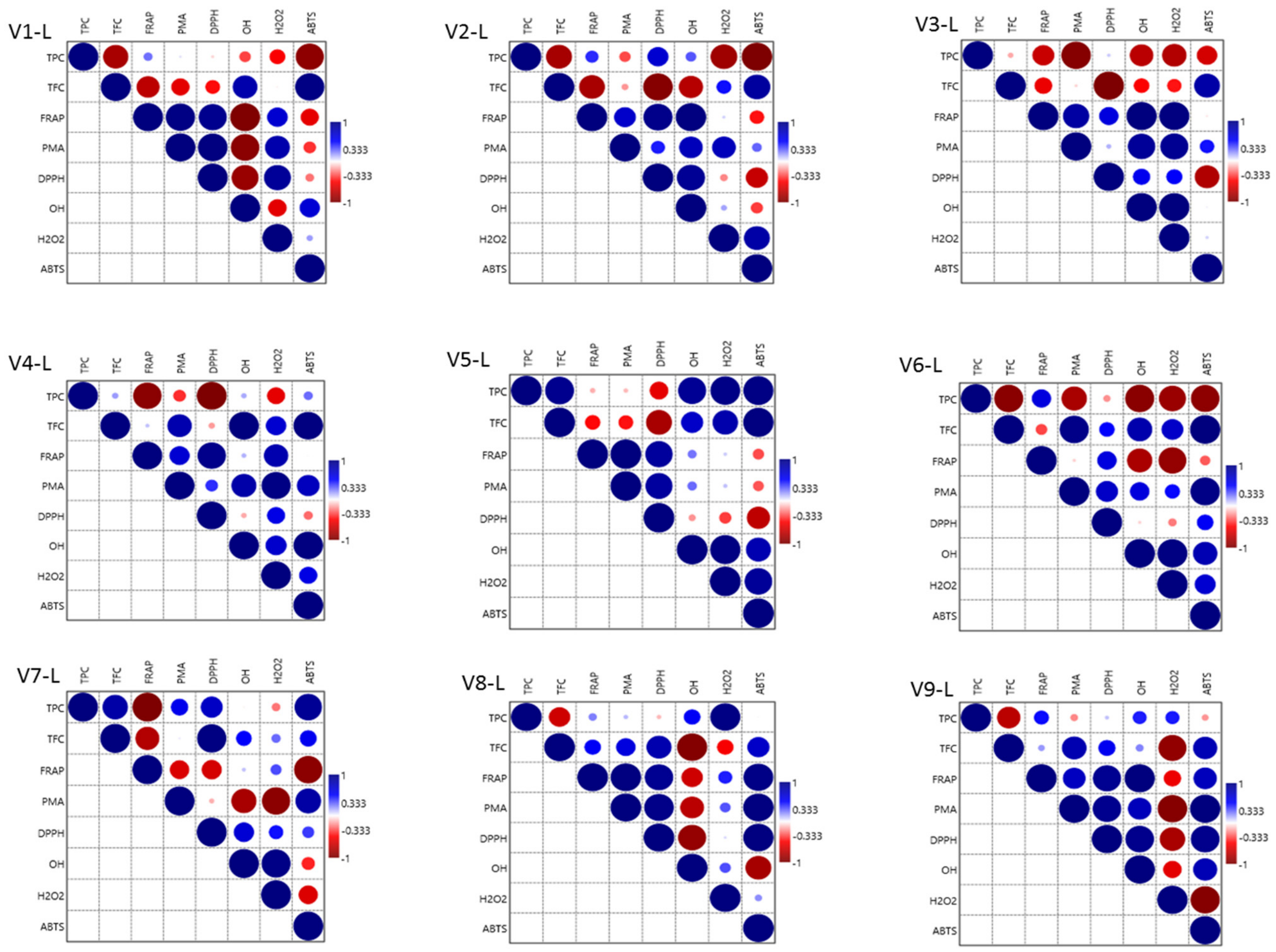

Figure S2. Correlation analysis between TPC, TFC and antioxidant activities in the leaves of onion varieties
